# A multi-parameter persistence framework for mathematical morphology

**DOI:** 10.1038/s41598-022-09464-7

**Published:** 2022-04-19

**Authors:** Yu-Min Chung, Sarah Day, Chuan-Shen Hu

**Affiliations:** 1 Eli Lilly and Company, Indianapolis, IN 46225 USA; 2 Department of Mathematics, William & Mary, Williamsburg, VA 23185 USA; 3 Department of Mathematics, National Taiwan Normal University, Taipei City, 106 Taiwan

**Keywords:** Applied mathematics, Computer science, Mathematics and computing

## Abstract

The field of mathematical morphology offers well-studied techniques for image processing and is applicable for studies ranging from materials science to ecological pattern formation. In this work, we view morphological operations through the lens of *persistent homology*, a tool at the heart of the field of topological data analysis. We demonstrate that morphological operations naturally form a multiparameter filtration and that persistent homology can then be used to extract information about both topology and geometry in the images as well as to automate methods for optimizing the study and rendering of structure in images. For illustration, we develop an automated approach that utilizes this framework to denoise binary, grayscale, and color images with salt and pepper and larger spatial scale noise. We measure our example unsupervised denoising approach to state-of-the-art supervised, deep learning methods to show that our results are comparable.

## Introduction

Digital image data is of importance in scientific applications ranging from materials science (e.g. micro-CT images of microstructure) to ecology (e.g. GIS or satellite images of plant and animal populations). Computational topology and the field of topological data analysis offer powerful tools for analyzing structure in data^1–5^. Persistent homology, in particular, has offered a means of measuring topological features across a *filtration*, or sequence of structures built from the data. A *one-filtration* is a collection of nested sets where the inclusion map enables the tracking of topological information from one set to the next. A *multifiltration* extends this notion to an indexed collection of sets satisfying an inclusion relation between a pair of sets whenever they are related under a specified partial order. This allows for the construction of many structures related to image or point cloud data, where appropriate inclusion relationships allow for the tracking of topological information across the structures. In this work, we use morphological operations to extend previous methods for filtration construction for digital images and use the resulting multiparameter filtrations for algorithmic exploration of image processing and denoising.

Mathematical morphology, a classic field in the image processing, provides theoretical and practical techniques for processing digital images^6–10^, such as smoothing, denosing, edge detection just to name a few. Operations in mathematical morphology are called the morphological operations. There are four fundamental morphological operations: erosion, dilation, opening, and closing. They are the building blocks of the mathematical morphology field and have many applications. One of them is to remove small scale features and smooth digital images^11,12^. These are well-developed operations for cleaning images by removing small scale features while keeping the remainder of the image relatively constant (see e.g. standard textbooks in mathematical morphology^13–15^. However, tracking changes of topological information with respect to parameters associated to those operations is not well-studied. Our focus in this work is to use multi-parameter persistent homology to tracks such topological changes.

Previous work in the field of topological data analysis has established one-filtrations for digital images and incorporated morphological operations in studies of height functions in Morse theory. In the cubical setting, a standard one-filtration for grayscale digital images is the sublevel set filtration obtained via thresholding. For example, in^16^, the authors build a cubical filtration by applying thresholding on probability distributions over image cubes. In Morse theory, one considers smooth functionals (called *height functions*) on a differentiable manifold to construct sublevel set filtrations^17,18^. Analogous construction works in the discrete case. For example, the authors in^19^ apply discrete Morse functions to build discrete Morse complexes, where the topology on generated Morse complexes can be used for analyzing tessellated manifolds. In^20^, the authors compute persistent homology of sublevel set filtrations induced from Morse functions on ${\mathbb {R}}^n$, providing an equivalence relation between persistent homology and morphological dynamics. They use this connection to investigate dynamical properties of Morse functions from a topological point of view. In^21^, the authors provide an interesting method for smoothing shapes of objects in 2 and 3-dimensional digital images, which preserves homotopy structure. To achieve this, the authors give the definition of homotopic equivalence of discrete images and construct homotopic thinning/thickening operations for shape smoothing. However, in image denoising tasks, one often aims to remove small scale features that arise due to noise in the image, thus sometimes dramatically changing the topology of the image. Therefore, in our approach, we do not impose homotopic equivalence and instead adopt a goal of intentionally changing the homological type of rendered structures in order to optimize topological and geometric accuracy by removing features most likely due to noise. Filtrations and persistence lend themselves well to automation. In^22^, Chung and Day used persistent homology to track structure in the sublevel set filtrations, developing an automated method for extracting topological measurements and thresholding grayscale images.

In this work, we focus on building an algebraic topological framework for the application of the morphological operations of erosion, dilation, opening, and closing. In order to track topological information for the resulting images, we construct new erosion (11), dilation (12), opening (13), and closing filtrations (14) and their variants  (15), (16), (17). We then show that under mild assumptions, combinations of these morphological operations, as well as thresholding, form multiparameter filtrations. This extends the use of multiparameter persistence from analyzing point cloud data in previous studies^23–28^, to now studying digital images. This approach also creates an algebraic topological formalism for combining and studying morphological operations, encoding the operations in a multifiltration framework with the goal of analyzing digital images in which features appear at different spatial scales. This includes noisy images in which the noise is smaller in spatial scale than the underlying structure we wish to uncover. We then demonstrate that it is possible to use this framework and persistent homology to extract information about underlying structure in the images as well as to automate the production of a denoised image. When combining morphological operations, the dimension of the constructed multiparameter filtration grows rapidly in the numbers of operations and utilized structuring elements (see “Application: a denoising algorithm for salt and pepper noise” section). Furthermore, thresholding may be combined with opening and closing to form a yet larger multifiltration for studying grayscale and, by extension, color images. As illustration, in “Application: a denoising algorithm for salt and pepper noise” section, we use opening, closing, and thresholding to construct a multifiltration. In order to demonstrate the utility of this framework, we develop a relatively simple algorithm that uses a directed walk through the filtration to remove small spatial scale (“salt and pepper”) noise and compare sample results for noisy binary, grayscale, and color images to those produced by existing supervised and unsupervised methods^29–31^.

In what follows, we introduce necessary definitions and properties for morphological operations (“Background on mathematical morphology” section) and persistent homology (“One‑parameter filtrations and persistent homology” section). “New filtrations based on morphological operations” section shows how to use morphological operations (erosion, dilation, opening, and closing) to construct one-parameter filtrations. We then use morphological operations and thresholding to build multifiltrations in “Multi‑parameter filtrations” section, presenting our main results in Theorem 1 and Corollary 1. This presents the necessary framework to combine morphological operations and, if appropriate, thresholding, in a single multiparameter filtration for analyzing digital data. As illustration, in “Application: a denoising algorithm for salt and pepper noise” section we combine opening, closing, and thresholding to construct a multiparameter filtration to analyze and denoise binary, grayscale, and color images with small scale (salt and pepper) noise.

## Background on mathematical morphology

In later sections, we will focus on using morphological operations to measure and track topological features in images. Here, we focus on establishing properties of the operations that are necessary to this topological approach. Two morphological operations that will be of particular interest in what follows are *dilation* and *erosion*. In a binary image, *dilation* enlarges features in the pixel subset for a specified value (e.g. 1, 255, or ‘black’) while *erosion* erases small, isolated features in the pixel subset (see^13–15^ and references therein).

We denote integers, natural numbers, and real numbers by the standard notation ${\mathbb {Z}}$, ${\mathbb {N}}$, and ${\mathbb {R}}$, respectively. We use ${\mathbb {Z}}_{\ge 0}$ to denote ${\mathbb {N}} \cup \{ 0\}$, the set of all non-negative integers. The symbol ${\mathbb {R}}_{\ge 0}$ represents the set of all non-negative real numbers. Elements in ${\mathbb {Z}}^m$ are denoted by boldface letters *e.g.* ${\mathbf {u}} \in {\mathbb {Z}}^m$ to distinguish vectors and scalars. We use this notation to build towards formalizing operations on digital images, which we define as follows.

### Definition 1

(Section 1.1.2.1^14^, p. 6) Let $m \in {\mathbb {N}}$ be a positive number, an *m-dimensional (digital) image* on *pixel/voxel set* $P\subseteq {\mathbb {Z}}^m$ is a non-negative function $g : P \rightarrow {\mathbb {R}}_{\ge 0}$. If the range of the function *g* is $\{0, 1\}$, then *g* is called a *binary image*. Otherwise, we refer to *g* as a *grayscale image*. The set of all images on *P*, denoted ${\mathscr {I}}_P$, is defined as ${\mathscr {I}}_P =\{g : P \rightarrow {\mathbb {R}}_{\ge 0}\}.$

**Figure 1:**
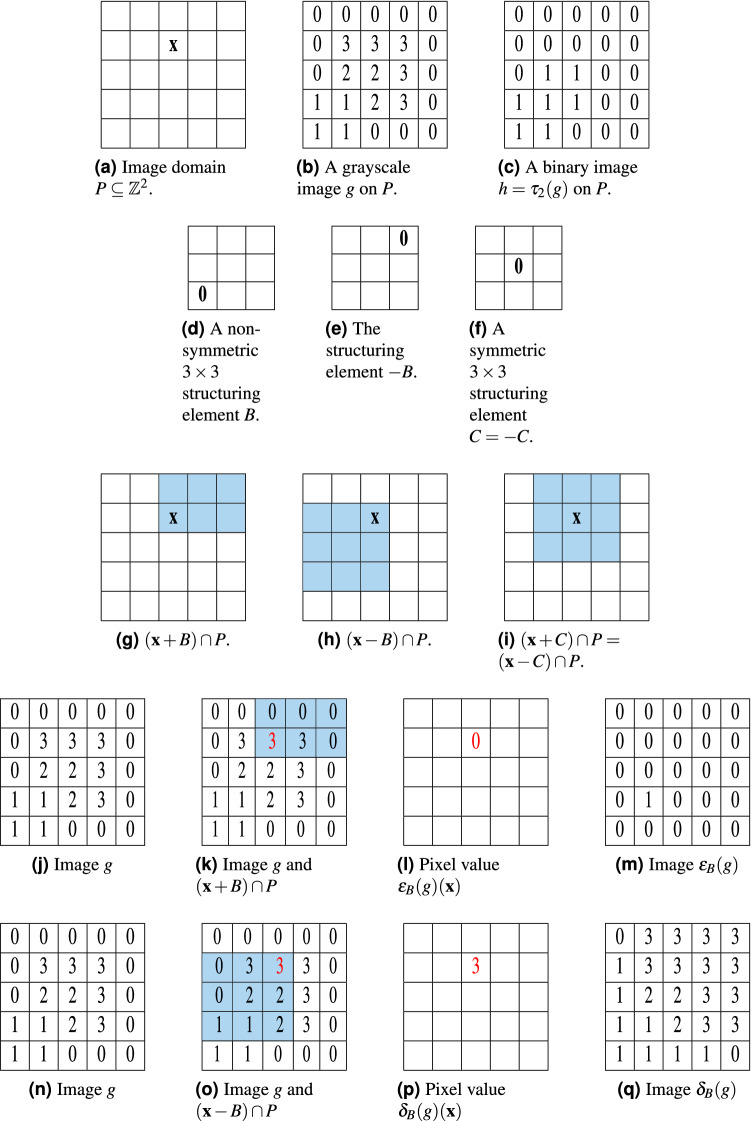
(**a**) A $5 \times 5$ image domain *P* lies in ${{\mathbb {Z}}}^2$, where ${{\mathbf {x}}}$ is a specified point in *P*. (**b**) A grayscale image is defined on the image domain *P*, it has pixel values 0, 1, 2,  and 3. (**c**) A binary image is defined on the image domain, where the pixels in the image with 0 are the black pixels and 1 for the white pixels. (**d**)–(**f**) Depictions of $3 \times 3$ structuring elements $B = \{ 0,1,2 \} \times \{ 0,1,2 \}$, $-B = \{ 0,-1,-2 \} \times \{ 0,-1,-2 \}$, and (symmetric) $C = \{ -1,0,1 \} \times \{ -1,0,1 \}$ with $\mathbf {0}=(0,0)$ marked. Blue regions in (**g**)–(**i**) are the sets $({{\mathbf {x}}}+ B) \cap P, ({{\mathbf {x}}}- B) \cap P$, $({{\mathbf {x}}}+ C) \cap P$, and $({{\mathbf {x}}}- C) \cap P$. Rows (**j**)–(**m**) and (**n**)–(**q**) are illustrations for erosion, dilation, opening, and closing operations.

**Figure 2:**
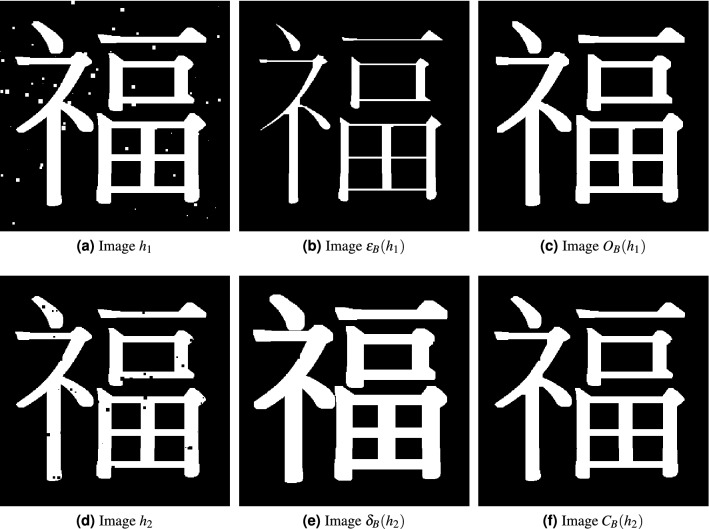
Illustration of erosion ($\varepsilon$), dilation ($\delta$), opening (*O*), and closing (*C*) with respect to the structuring element $B_6$ which is a $7\times 7$ square defined in (10).

**Figure 3:**
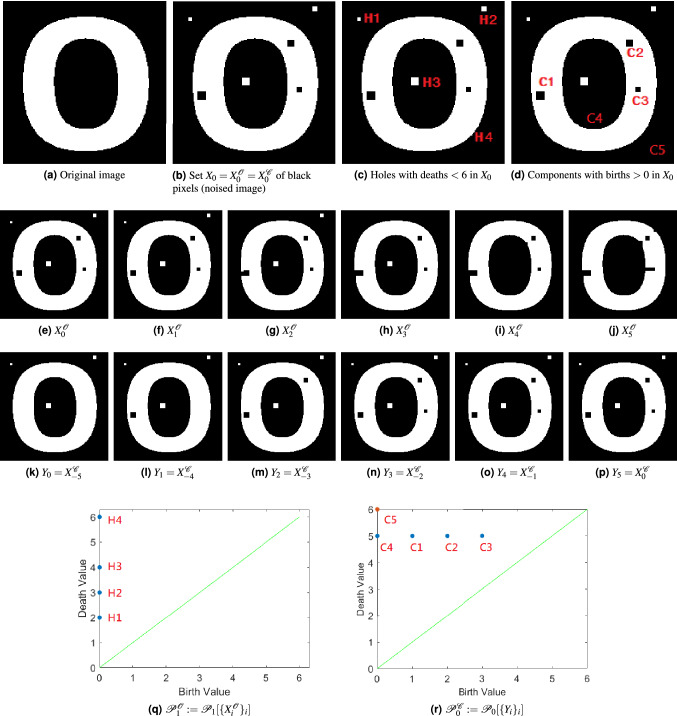
Opening filtration $X^{{\mathscr {O}}}_i := O_{{\mathfrak {B}}_i}(f)^{-1}(0)$ (row 2), closing filtration $Y_i := X^{{\mathscr {C}}}_{-(5 - i)} := C_{{\mathfrak {B}}_i}(f)^{-1}(0)$ (row 3), and their relevant persistence diagrams (row 4). (**a**) Original binary image *g*; (**b**) noised image *f*; (**c**) the 1-dimensional holes in $X_0$ that have death $< 6$; (**d**) the connected components in $X_0$ that have birth $> 1$; (**e**)–(**j**) binary representations of $X^{{\mathscr {O}}}_i := O_{{\mathfrak {B}}_i}(f)^{-1}(0)$ with $i = 0, 1, \ldots , 5$; (**k**)–(**p**) binary representations of $Y_i := X^{{\mathscr {C}}}_{-(5 - i)} := C_{{\mathfrak {B}}_i}(f)^{-1}(0)$ with $i = 0, 1, \ldots , 5$. The structuring elements ${\mathfrak {B}}_0, {\mathfrak {B}}_1, ..., {\mathfrak {B}}_5$ used here are the $B_0, B_2, B_4, B_6, B_8$, and $B_{10}$ in (10). (**q**) Persistence diagram ${\mathscr {P}}_1[\{X^{{\mathscr {O}}}_i\}_{i=0}^{5}]$. (**r**) Persistence diagram ${\mathscr {P}}_0[\{Y_i\}_{i=0}^{5}]$. By diagrams (**q**) and (**r**), for the image in (**b**), the values $i_o$ and $i_c$ derived from the first iteration in Algorithm 1 are 2 and $(5 + 1) - 3 = 3$ respectively.

In practice, one usually considers a rectangular image whose domain can be expressed as

$$\begin{aligned} P = {{\mathbb {Z}}}^m \cap \left( \prod _{i = 1}^m [a_i, b_i] \right) = \{ a_1, a_1+1, ..., b_1 \} \times \cdots \times \{ a_m, a_m+1, ..., b_m \} \end{aligned}$$

where $a_i \le b_i$ are integers. Figure 1a is an illustration for the $5 \times 5$ image domain *P* in ${{\mathbb {Z}}}^2$ (*e.g.* $P = \{ 0,1, ..., 4\} \times \{ 0,1, ..., 4\}$). Figure 1b,c are examples of grayscale and binary images defined on *P*.

The following discussion focuses primarily on binary images $g : P \rightarrow \{ 0, 1\}$, where a value of 1 means the pixel is white and a value of 0 means the pixel is black, and 8-bit grayscale images $g:P \rightarrow \{ 0,1, ..., 255\}$ where 225 is white and 0 is black. When feasible, we consider general images $g : P \rightarrow {\mathbb {R}}_{\ge 0}$ so that properties and theorems stated in the paper hold in this general case. This setting is also convenient when considering re-scaling of pixel values of images with different range sets.

We now establish the following partial order on the space of images and define image and preimage subsets. Given two images $f, g : P \rightarrow {\mathbb {R}}$, we say that $f \le g \text { if and only if } f({{\mathbf {p}}}) \le g({{\mathbf {p}}})~ \text { for all } {{\mathbf {p}}}\in P$. For functions $f : S \rightarrow T$, $A \subseteq S$ and $B \subseteq T$, the sets $f(A) := \{ f(a) \ | \ a \in A \}$ and $f^{-1}(B) := \{ s\in S \ | \ f(s) \in B \}$ are the *image of A* and the *preimage of B* under *f*. As an abbreviation, if $B = \{ b \}$ is a singleton set, we write $f^{-1}(b)$ instead of $f^{-1}(\{b \})$ to denote $f^{-1}(B)$.

### Definition 2

(Section 3.1^13^ p. 64). For $m \in {\mathbb {N}}$, a *structuring element* is a specified finite set *B* satisfying ${\mathbf {0}} \in B \subseteq {\mathbb {Z}}^m$. A structuring element *B* is *symmetric* if $B = -B := \{ -{{\mathbf {x}}}\ | \ {{\mathbf {x}}}\in B \}$.

Figure 1d shows an example of a structuring element which is a square defined as $B = \{0,1,2\}\times \{0,1,2\}$. It is important to note that the origin (0, 0) is in the structuring element *B*. In mathematical morphology, the reflection of the structuring element over the origin is often used. Figure 1e shows the reflection of *B*, i.e. $-B =\{0,-1,-2\}\times \{0,-1,-2\}$. We observe that $B\ne -B$ in Fig. 1d,e, giving examples of *non-symmetric* structuring elements. (If a structuring element is equal to its reflection, it is called *symmetric*.) Figure 1f shows an example of a symmetric structuring element.

### Remark 1

In mathematical morphology, structuring elements defined here are called *flat* structuring elements^13^. In certain applications, a *non-flat* structuring element ${\mathscr {B}}$ is defined as a function from a finite subset *B* of ${{\mathbb {Z}}}^m$ to ${{\mathbb {R}}}_{\ge 0}$ which records weighted values for elements in *B*. In this case, a flat structuring element can be viewed as a characteristic function $\chi _B$ on a finite set $B \subseteq {{\mathbb {Z}}}^m$. In this paper, all structuring elements we consider are flat.

Structuring elements will be used to define local windows over which pixel values are considered during processing operations. This requires the *Minkowski sum* and *difference* for subsets $A,~B \subseteq {\mathbb {Z}}^m$, defined respectively as

$$\begin{aligned} \begin{aligned} A + B&:= \{ {{\mathbf {a}}}+ {{\mathbf {b}}}\ | \ {{\mathbf {a}}}\in A, {{\mathbf {b}}}\in B \}, \\ A - B&:= \{ {{\mathbf {a}}}- {{\mathbf {b}}}\ | \ {{\mathbf {a}}}\in A, {{\mathbf {b}}}\in B \}. \end{aligned} \end{aligned}$$

If either $A = \{ {{\mathbf {a}}}\}$ or $B = \{ {{\mathbf {b}}}\}$ are sets of singleton points, we would simply use ${{\mathbf {a}}}+ B$, ${{\mathbf {a}}}- B$, $A + {{\mathbf {b}}}$ or $A - {{\mathbf {b}}}$ rather than $\{ {{\mathbf {a}}}\} + B$, $\{ {{\mathbf {a}}}\} - B$, $A + \{ {{\mathbf {b}}}\}$ or $A - \{ {{\mathbf {b}}}\}$ to denote the Minkowski sum or difference of *A* and *B*s. Consider ${{\mathbf {x}}}+ B$ where ${{\mathbf {x}}}= (2,3) \in P$ and $B = \{0,1,2\}^2$. By the above definition of Minkowski sum, we obtain that ${{\mathbf {x}}}+ B = \{(2,3)\} + \{(0,0),(1,0), (2,0), (0,1), (1,1), (2,1), (0,2), (1,2), (2,2)\} = \{ (2,3), (3,3), (4,3), (2,4), (3,4), (4,4), (2,5), (3,5), (4,5) \}$. (See also Fig. 1.)

Since $\mathbf{0} \in B$, one may think of ${{\mathbf {x}}}+B$ as the *B*-*neighborhood* of ${{\mathbf {x}}}$ in ${\mathbb {Z}}^m$. If $g \in {\mathscr {I}}_P$ is a binary image and $g({{\mathbf {x}}}+ B) = \{ 1 \}$, then the *B*-neighborhood of ${{\mathbf {x}}}$ is contained in the white region of the image. On the other hand, if $g({{\mathbf {x}}}+ B) = \{ 0, 1 \}$, then the *B*-neighborhood of ${{\mathbf {x}}}$ intersects both the white and the black sets in the image.

In this work, we consider four fundamental morphological operations: *erosion*, *dilation*, *opening*, and *closing*. We next review their formal definitions.

### Definition 3

(Equations (1.6) and (1.7)^14^ p. 10) For $g \in {\mathscr {I}}_P$ and structuring element $B \subseteq {\mathbb {Z}}^m$, the *erosion* of *g* via *B* is an image $\varepsilon _B(g) \in {\mathscr {I}}_P$ defined by

1 $$\begin{aligned} \varepsilon _B(g)({{\mathbf {x}}}) = \min g\bigg (({{\mathbf {x}}}+ B) \cap P\bigg ) = \min \{ g({{\mathbf {x}}}+{{\mathbf {b}}}) \ | \ {{\mathbf {b}}}\in B, {{\mathbf {x}}}+{{\mathbf {b}}}\in P \}. \end{aligned}$$

Similarly, the *dilation* of *g* via *B* is an image $\delta _B(g) \in {\mathscr {I}}_P$ defined by

2 $$\begin{aligned} \delta _B(g)({{\mathbf {x}}}) = \max g\bigg (({{\mathbf {x}}}- B) \cap P\bigg ) = \max \{ g({{\mathbf {x}}}-{{\mathbf {b}}}) \ | \ {{\mathbf {b}}}\in B, {{\mathbf {x}}}-{{\mathbf {b}}}\in P \}. \end{aligned}$$

Since ${\mathbf {0}} \in B$ and *B* is finite, $({\mathbf {x}} + B) \cap P$ and $({\mathbf {x}} - B) \cap P$ are non-empty, finite sets whenever ${{\mathbf {x}}}\in P$. Therefore, $\varepsilon _B(g)$ and $\delta _B(g)$ are well-defined.

The intersection with the image domain *P* is needed in Definition 3 because $({{\mathbf {x}}}+ B)$ can be beyond the boundary of *P*. For example, we demonstrated above that ${{\mathbf {x}}}+ B = \{(2,3)\} + \{(0,0),(1,0), (2,0), (0,1), (1,1), (2,1), (0,2), (1,2), (2,2)\} = \{ (2,3), (3,3), (4,3), (2,4), (3,4), (4,4), (2,5), (3,5), (4,5) \}$, where *P* and *B* are depicted in Fig. 1a,d, respectively. Clearly, some elements of ${{\mathbf {x}}}+B$ are not in *P*, e.g. $(2,5) \not \in P$. Examples of $({{\mathbf {x}}}+ B) \cap P$ and $({{\mathbf {x}}}- B) \cap P$ are shown in Fig. 1g,i. As shown in Definition 3, these sets operation are essential for $\varepsilon _B(g)({{\mathbf {x}}})$ and $\delta _B(g)({{\mathbf {x}}})$. Figure 1k–m demonstrate steps to obtain $\varepsilon _B(g)({{\mathbf {x}}})$ where *g* is given and shown in Fig. 1j. Figure 1k shows the set $({{\mathbf {x}}}+ B) \cap P$ where the point $g({{\mathbf {x}}})$ is colored as red and their pixel values $g({{\mathbf {x}}}+ B)$ are shaded in the blue color which consist of 0, 0, 0, 0, 3, and 3. Since the minimum value of those blue shaded values is 0, $\varepsilon _B(g)({{\mathbf {x}}}) = 0$ as shown in Fig. 1l. Repeat this process for all ${{\mathbf {x}}}$ and the final processed image is shown in Fig. 1m Similarly, Fig. 1o,q demonstrate steps to obtain $\delta _B(g)({{\mathbf {x}}})$ where *g* is given and shown in Fig. 1n. Figure 1o shows the set $({{\mathbf {x}}}- B) \cap P$ where the point $g({{\mathbf {x}}})$ is colored as red and their pixel values $g({{\mathbf {x}}}+ B)$ are shaded in the blue color which consist of 0, 3, 3, 0, 2, 2, 1, 1, and 2. Since the maximum value of those blue shaded values is 3, $\delta _B(g)({{\mathbf {x}}}) = 3$ as shown in Fig. 1p. Repeat this process for all ${{\mathbf {x}}}$ and the final processed image is shown in Fig. 1q.

### Remark 2

Observe that if $B \subseteq {{\mathbb {Z}}}^m$ is symmetric, then (2) is equivalent to

3 $$\begin{aligned} \delta _B(g)({{\mathbf {x}}}) = \max g\bigg (({{\mathbf {x}}}+ B) \cap P\bigg ) = \max \{ g({{\mathbf {x}}}+{{\mathbf {b}}}) \ | \ {{\mathbf {b}}}\in B, {{\mathbf {x}}}+{{\mathbf {b}}}\in P \}. \end{aligned}$$

### Remark 3

Dilation and erosion operations in Definition 3 are commonly adopted in the mathematical morphology^13,14,32^. Recently, the work^33^ uses the terms dilation and erosion, but their definitions and meanings are different than the classic ones. For example, “dilation” $\Delta$ in^33^ is defined as follows: for a binary image $g : P \rightarrow \{ 1, 0 \}$ and ${\mathbf {x}} \in P$,

$$\begin{aligned} \Delta (g)({{\mathbf {p}}}) := \min \{ \Vert {{\mathbf {p}}}- {{\mathbf {x}}}\Vert _1 : {{\mathbf {x}}}\in P, \ g({{\mathbf {x}}}) = 1 \}. \end{aligned}$$

This operation is referred to as the *distance transformation* in other texts on mathematical morphology^13,14,32^.

Erosion and dilation serve as the “building blocks” of mathematical morphology and many other morphological operations are defined as compositions of erosion and dilation. Two examples are *opening* and *closing*. These operations are commonly used for smoothing images.

### Definition 4

(Section 1.2.1^14^, p. 12) Let $P,B\subseteq {\mathbb {Z}}^m$ be a pixel set and structuring element respectively. Then *opening* and *closing* operations via *B*, denoted by $O_B$ and $C_B$ respectively, are functions $O_B, C_B: {\mathscr {I}}_P \rightarrow {\mathscr {I}}_P$ defined as

4 $$\begin{aligned} O_{B} = \delta _{B}\,{^\circ }\, \varepsilon _{B} \;\;and\;\;C_{B} = \varepsilon _{B}\,{^\circ } \,\delta _{B} . \end{aligned}$$

Figure 2 visualizes the processed images by erosion, dilation, opening, and closing operator. Intuitively speaking, erosion and opening tend to shave off white regions in an image. On the other hand, dilation and closing tend to shave off black regions in an image. Observe that Fig. 2a contains small isolated white squares while Fig. 2d contains small isolated black squares. Applying the erosion operator with respect to $B_6$ to Fig. 2a results in Fig. 2b. Comparing Fig. 2a,b, we observe that small white squares are removed and since too much white regions are removed, the resultant kanji character becomes “thinner” as shown in Fig. 2b. Opening operation, which is erosion followed by dilation, accounts for such “loss” as shown in Fig. 2c. Similarly, as shown in Fig. 2d–f, the image $h_2$ contains small black squares, the dilation $\delta _B(h_2)$ would remove them and thicken the overall white regions, and the closing $C_B(h_2)$ would better preserve the structure. For opening and closing operation, not only the small noises are removed but also the overall kanji character is kept well. This is the reason opening and closing are commonly used tools for image filtering (see e.g. the classic textbooks^13–15^).

The opening and closing operations may be used to remove structure that is smaller than the scale prescribed by *B* while minimizing distortion of larger scale features^13,34–36^. It is clear that if $B = \{ {\mathbf {0}} \}$, then $\varepsilon _{B} = \delta _{B} = {\text{id}}_{{{\mathscr{I}}_{P} }}$ where $\mathrm{id}_{{\mathscr {I}}_P} : {\mathscr {I}}_P \rightarrow {\mathscr {I}}_P$ denotes the identity function and $O_B = C_B = \mathrm{id}_{{\mathscr {I}}_P}$.

We conclude this section by reviewing some basic properties of these morphological operations. We will use these properties to establish our main result.

### Proposition 1

(Properties 3.4^13^, p. 71) Let $f, g \in {\mathscr {I}}_P$ be images and $B \subseteq {\mathbb {Z}}^m$ be a structuring element. If $f \le g$, then the inequalities $\delta _{B} (f) \le \delta _{B} (g),~\,\, \varepsilon _{B} (f) \le \varepsilon _{B} (g),~\,\,O_{B} (f) \le O_{B} (g),~\,\,{\text{and}}\,\,{\text{ }}C_{B} (f) \le C_{B} (g)$ hold.

Proposition 1 states the *increasing property*, that is, for a fixed structuring element, the basic morphological operations preserve the ordering relation on images. In Definition 1, we define images as functions. Our main focus in this work is to construct a *filtration* or collection of sets ordered by set inclusion. We do this for image sublevel sets, i.e. subsets of the pixel set *P* corresponding to pixels with image values at or below a prescribed threshold value. When we consider binary images, the filtration property of sublevel sets is naturally related to the increasing property as shown in the following proposition.

### Proposition 2

(Principle 11.1.1^13^, p. 318). Let $f, g \in {\mathscr {I}}_P$ be images. If $f \le g$, then $g^{-1}(0) \subseteq f^{-1}(0)$. In addition, if $f, g : P \rightarrow \{ 0,1 \}$ are binary images, then $f \le g$ if and only if $g^{-1}(0) \subseteq f^{-1}(0)$ and, similarly, $f \le g$ if and only if $f^{-1}(1) \subseteq g^{-1}(1)$.

The dilation and erosion operators are defined by a given structural element. We are also interested in the increasing property of dilation and erosion operators. More precisely, given two structural elements with subset relation, their corresponding dilation and erosion follow certain subset relations.

### Proposition 3

If $B_1 \subseteq B_2 \subseteq {\mathbb {Z}}^m$ be structuring elements, then $\delta _{{B_{1} }} (g) \le \delta _{{B_{2} }} (g){\text{,}}\,\,{\text{ and}}\,\,{\text{ }} \varepsilon _{{B_{2} }} (g) \le \varepsilon _{{B_{1} }} (g)$ for every $g \in {\mathscr {I}}_P$.

The proof of Proposition 3 can be derived directly from the Definition 3. There are many ways to produce a binary image from a grayscale image. *Global thresholding* of grayscale image $g:P\rightarrow {\mathbb {R}}_{\ge 0}$ via threshold value *t* produces the binary image

5 $$\begin{aligned} g_{t}({{\mathbf {x}}}) = {\left\{ \begin{array}{ll} 0 \quad \text { if } g({{\mathbf {x}}}) \le t, \\ 1 \quad \text { otherwise.} \end{array}\right. } \end{aligned}$$

Note that $g_t^{-1}(0)=\{{{\mathbf {x}}}\varepsilon P:g({{\mathbf {x}}})\le t\}$. This set, $g_t^{-1}(0)$ is the *t*-*sublevel set* of *g*. In general, the operations of erosion, dilation, opening, and closing do not commute. However, these four operations do commute with the operation of global thresholding as follows.

### Proposition 4

(Proposition 1^37^) For $m \varepsilon {\mathbb {N}}$ and pixel set $P \subseteq {{\mathbb {Z}}}^m$, consider the image $g \in {\mathscr {I}}_P$. For each threshold $t \in {\mathbb {R}}_{\ge 0}$ we define $\tau _t : {\mathscr {I}}_P \rightarrow {\mathscr {I}}_P$ by $g \mapsto g_t$ i.e.,

6 $$\begin{aligned} \tau _t(g)({{\mathbf {x}}}) = g_{t}({{\mathbf {x}}}) = {\left\{ \begin{array}{ll} 0 \quad \text { if } g({{\mathbf {x}}}) \le t, \\ 1 \quad \text { otherwise.} \end{array}\right. }. \end{aligned}$$

For any structuring element $B \subseteq {\mathbb {Z}}^m$, the following diagrams are commutative:i.e., $\varepsilon _{B}\, \circ\, \tau _{t} = \tau _{t}\,\circ \,\varepsilon _{B}$ and $\delta _B \circ \tau _t = \tau _t \circ \delta _B$. Moreover, by combining these two commutative diagrams, $O_B \circ \tau _t = \tau _t \circ O_B \ \ {and} \ \ C_B \circ \tau _t = \tau _t \circ C_B.$

## One-parameter filtrations and persistent homology

In this section, we show that the partial order results for morphological operations presented in “Background on mathematical morphology” section naturally yield the structure necessary for computing *persistent homology*.

Persistent homology, a foundational tool in the field of topological data analysis (TDA), measures and tracks topological features. It relies on having a *one-parameter filtration*, a sequence of nested sets. The goal of this section is to introduce two new filtrations based on morphological operations and define and illustrate the meaning of *persistence diagrams* based on these filtrations.

Topological features of interest include connected components (or individual connected pieces of the set), one-dimensional holes (holes in 2d or tunnels in 3d) and two-dimensional holes (cavities in 3d). Higher dimensional holes may appear in higher dimensional data, but for illustration, we will focus on two and three dimensional data sets here. The framework we present throughout this work applies to higher dimensional data and holes as well. For the data sets we study, cubical homology may be summarized using Betti numbers. Betti numbers, $\beta _k$, count holes of various dimensions. More specifically, given a binary image *f*, if we consider the set of black pixels, $X:=f^{-1}(0)$, then $\beta _0(X)$ is the number of connected components, $\beta _1(X)$ is the number of 1-dimensional holes, or tunnels, $\beta _2(X)$ is the number of 2-dimensional holes, or cavities, etc. They are computed using algebraic structure defined by the cubical structure of *X* and there are now efficient software packages for performing these calculations. See, for example^38^, and references therein for a discussion of the mathematical theory behind the definition and computation of Betti numbers as well as their interpretation as direct counts of topological features.

Persistent homology extends the topological measurement offered by Betti numbers across a filtration. A *one-parameter filtration* is a sequence of sets $\{X_i\}_{i\in A}$, with indexing set $A\subset {\mathbb {Z}}$, satisfying

7 $$\begin{aligned} X_i \subseteq X_j, \quad \text {whenever } i\le j. \end{aligned}$$

For ease of notation, we will often write $\{X_i\}$ when the indexing set has already been specified. For a one-parameter filtration, persistent homology records the *birth* and *death* coordinates at which a given topological feature first appears and first disappears respectively in cubical sets. That is, given a one-parameter filtration $\{X_i\}_{i\in A}$ of cubical sets, a feature with birth/death coordinates (*b*, *d*), does not exist in the sets $X_i$ with $i<b$, appears first in $X_b$ and persists through all sets $X_j$ with $b\le j < d$ and disappears in $X_{d}$. Like Betti numbers, birth/death coordinates are computed using algebraic structure attached to cubical sets, in this case using the inclusion operation to match some features in $X_n$ to their preimages in $X_{n-1}$.

The collection of birth/death pairs for all topological features, labeled by dimension of the feature, is called a *persistence diagram*. For a given one-parameter filtration $\{X_i\}$, the full persistence diagram is the multiset of all birth/death pairs and is denoted by $\mathscr{{P}}(\{X_i\})$, with the *k-th persistence diagram*, $\mathscr{{P}}_k(\{X_i\})$, denoting the subset of pairs measuring *k*-dimensional holes. Note that different hole structures may have the same persistence interval (*b*, *d*). The persistence diagram is a multiset since any birth/death coordinate (*b*, *d*) can appear multiple times. By this convention, $\mathscr{{P}}(\{X_i\}) = \displaystyle \cup _{k}\mathscr{{P}}_k(\{X_i\})$. For ease of notation, we represent these persistence diagrams by $\mathscr{{P}}$ (respectively $\mathscr{{P}}_k$) when the filtration $\{X_i\}$ is understood. Betti numbers may be extracted from persistence diagrams as

8 $$\begin{aligned} \beta _k( X_m ) = \#\mathscr{{P}}_k(m), \end{aligned}$$

where

$$\begin{aligned} \mathscr{{P}}_k(m) := \{(b,d)\in \mathscr{{P}}_k \mid \; b\le m,\; d>m\}. \end{aligned}$$

In other words, $\beta _k( X_m )$ counts the number of points in the k-th persistence diagram whose birth/death coordinates indicate that they are present in set $X_m$. By extension, we also write $\mathscr{{P}}(m):=\cup _k \mathscr{{P}}_k(m)$. Furthermore, a given feature’s lifespan, $l=d-b$, measures the length of the interval of set indices over which the feature persists. Birth/death coordinates and corresponding lifespans allow us to study the robustness of the feature with respect to changes in the index *m*.

For more information about persistent homology, see e.g.^2,4^, and references therein. In summary, given a one-parameter filtration of cubical sets, persistence diagrams are efficient to compute. The reference^39^ provides an overview of current TDA software. In this work, we use Perseus^40^ and DIPHA^41^ for cubical persistent homology computations.

Researchers have developed several methods for creating one-parameter filtrations. The most fundamental and commonly-used filtration for grayscale digital images is the *sublevel set filtration* (see, e.g.^33,42^). Using the sublevel set and thresholding operations (6), for thresholds $t_1 \le t_2 \le \cdots \le t_n$,

9 $$\begin{aligned} g^{-1}_{t_1}(0) \subseteq g^{-1}_{t_2}(0) \subseteq \cdots g^{-1}_{t_n}(0). \end{aligned}$$

Setting $X_i=g^{-1}_{t_i}(0)$ yields a sublevel set filtration $\{X_i\}$. Cubical homology and sublevel set filtrations have been used to study images. For example, in^16^, the authors develop an image segmentation algorithm by incorporating persistence diagrams for sublevel set filtrations on cubical sets into deep learning architecture. For binary images, there are techniques for constructing related grayscale images that would then lead to sublevel set filtrations. These include, for example, using a signed distance function or density estimator to define grayscale values^33,43^. While^44^ considers the changes of size functions (the 0-th persistence diagram of the sublevel set filtration) under the skeleton operation which combines certain morphological operations, our goal here is build a general filtration framework using erosion, dilation, opening, and closing. To the best of our knowledge, the proposed work is the first to use morphological operations and thresholding to construct a filtration directly.

## New filtrations based on morphological operations

We now use morphological operations to form new filtrations for binary and grayscale images. For ease of discussion, throughout the article we use a sequence of structuring elements, $\{B_i\}_{i=0}^n$, where each $B_i$ is a $(i+1)\times (i+1)$ square given by

10 $$\begin{aligned} \begin{aligned} B_0&= \{ (0,0) \},\\ B_n&= \left\{ \begin{array}{ll} B_{n-1} \cup (B_{n-1} + {{\mathbf {e}}}_1) \cup (B_{n-1} + {{\mathbf {e}}}_2) \cup (B_{n-1} + {{\mathbf {e}}}_1 + {{\mathbf {e}}}_2) &{} \text{ if } \text{ n } \text{ is } \text{ odd }\\ B_{n-1} \cup (B_{n-1} - {{\mathbf {e}}}_1) \cup (B_{n-1} - {{\mathbf {e}}}_2) \cup (B_{n-1} - {{\mathbf {e}}}_1 - {{\mathbf {e}}}_2) &{} \text{ if } \text{ n } \text{ is } \text{ even, } n \ge 2 \end{array}\right. , \end{aligned} \end{aligned}$$

where ${{\mathbf {e}}}_1 = (1,0)$ and ${{\mathbf {e}}}_2=(0, 1)$. These may be depicted aswhere ${\mathbf {0}}$ represents the origin $(0,0) \in {\mathbb {Z}}^2$. Clearly, $B_0\subseteq B_1 \subseteq \cdots \subseteq B _n$. Note that since $B_0=\{(0,0)\}$, the erosion/dilation, and opening/closing operations with respect to $B_0$ are the identity map.

Other sequences of structuring elements will also give rise to filtrations. In particular, there is a notion of *shift inclusion* that may be used to designate a large class of sequences of structuring elements that may be used to form filtrations. That topic is studied in detail in^37^.

The first new filtrations we propose are for binary images and use the erosion and dilation operations. We consider a sequence of erosion and dilation operations with respect to $\{B_i\}_{i=0}^n$, i.e. for each *i*, consider $\delta _{B_i}(f)$ and $\varepsilon _{B_i}(f)$ for a given binary image *f*. Similar to the sublevel set filtration in (9), the desired property is that if $i\le j$ ($B_i \subseteq B_j$), then $\varepsilon _{B_i}(f)^{-1}(0) \subseteq \varepsilon _{B_j}(f)^{-1}(0)$. Thanks to Proposition 3, it is straightforward to verify that

11 $$\begin{aligned}&\varepsilon _{B_0}(f)^{-1}(0) \subseteq \varepsilon _{B_1}(f)^{-1}(0) \subseteq \cdots \subseteq \varepsilon _{B_n}(f)^{-1}(0), \end{aligned}$$

12 $$\begin{aligned}&\delta _{B_n}(f)^{-1}(0) \subseteq \delta _{B_{n-1}}(f)^{-1}(0) \subseteq \cdots \subseteq \delta _{B_0}(f)^{-1}(0). \end{aligned}$$

This shows that for any sequence of nested structuring elements, erosion and dilation form filtrations. We call $\{X^{\varepsilon }_i\}_{i=0}^n$, where $X^{\varepsilon }_i=\varepsilon _{B_i}(f)^{-1}(0)$, the *erosion filtration*, and $\{X^{\delta }_j\}_{j=-n}^0$, where $X^{\delta }_j=\delta _{B_{|j|}}(f)^{-1}(0)$, the *dilation filtration*. Note that since $\varepsilon _{B_0}(f)^{-1}(0) = f^{-1}(0) = \delta _{B_0}(f)^{-1}(0)$, we may form one extended filtration by taking $\{\tilde{X}_i\}_{i=-n}^n$, where for $i<0$, $\tilde{X}_i= X^{\delta }_i$, and for $i>0$, $\tilde{X}_i= X^{\varepsilon }_i$.

The second new filtrations we propose is related to the opening and closing operations. Since opening and closing are compositions of erosion and dilation operations, one may expect that Proposition 3 would extend to the case of opening or closing. However, it is not true in general. We refer readers to^37^ for a counter example and more discussion. Essentially, the sequence of structuring elements cannot be arbitrary and requires additional assumptions^37^. Presents a sufficient condition called *shift inclusion* that guarantees the structure necessary for opening and closing to result in appropriately nested sets.

Since our chosen square structuring elements, $B_i$, satisfy shift inclusion^37^, $O_{B_i}$ and, separately, $C_{B_i}$, also form filtrations.

13 $$\begin{aligned}&O_{B_0}(f)^{-1}(0) \subseteq O_{B_1}(f)^{-1}(0) \subseteq \cdots \subseteq O_{B_n}(f)^{-1}(0); \end{aligned}$$

14 $$\begin{aligned}&C_{B_n}(f)^{-1}(0) \subseteq C_{B_{n-1}}(f)^{-1}(0) \subseteq \cdots \subseteq C_{B_0}(f)^{-1}(0). \end{aligned}$$

Similar to erosion and dilation filtration, we call $\{X^{{\mathscr {O}}}_i\}_{i=0}^n$, where $X^{{\mathscr {O}}}_i= O_{B_i}(f)^{-1}(0)$, the *opening filtration*, and $\{X^{{\mathscr {C}}}_j\}_{j=-n}^0$, where $X^{{\mathscr {C}}}_j=C_{B_{|j|}}(f)^{-1}(0)$, the *closing filtration*. Note that since $O_{B_0}(f)^{-1}(0) = f^{-1}(0) = C_{B_0}(f)^{-1}(0)$, we may form one extended filtration by taking $\{\tilde{X}_i\}_{i=-n}^n$, where for $i<0$, $\tilde{X}_i= X^{{\mathscr {C}}}_i$, and for $i>0$, $\tilde{X}_i= X^{{\mathscr {O}}}_i$.

As a byproduct, applications of (13) and (14) lead to three additional filtrations based on the commonly used top-hat transformation: the *white top hat* $\text {WTH}_B(f) = f - O_B(f)$, the *black top hat* $\text {BTH}_B(f) = C_B(f) - f$, and the *self complementary top hat transformation* $\text {STH}_B(f) = C_B(f) - O_B(f)$^13,15,45^. More precisely, one has

15 $$\begin{aligned}&\text {WTH}_{B_n}(f)^{-1}(0) \subseteq \text {WTH}_{B_{n-1}}(f)^{-1}(0) \subseteq \cdots \subseteq \text {WTH}_{B_0}(f)^{-1}(0), \end{aligned}$$

16 $$\begin{aligned}&\text {BTH}_{B_n}(f)^{-1}(0) \subseteq \text {BTH}_{B_{n-1}}(f)^{-1}(0) \subseteq \cdots \subseteq \text {BTH}_{B_0}(f)^{-1}(0), \end{aligned}$$

17 $$\begin{aligned}&\text {STH}_{B_n}(f)^{-1}(0) \subseteq \text {STH}_{B_{n-1}}(f)^{-1}(0) \subseteq \cdots \subseteq \text {STH}_{B_0}(f)^{-1}(0). \end{aligned}$$

Figure 3 depicts examples of opening and closing filtrations and two of their resulting persistence diagrams. Figure 3a shows the original binary image, *f*, and collection of black pixels $X_0 := f^{-1}(0)$. We observe that there are five connected components (disjoint black pieces) depicted as C1, C2, C3, C4, and C5 in Fig. 3d and there are 4 holes (isolated white regions) depicted as H1, H2, H3, and H4 in Fig. 3c. Thus, $\beta (X_0) = (5, 4)$. We also observe that the spatial sizes of features C1–C5 and H1-H4 are different but each contribute equally (is counted once) to the overall Betti numbers. However, the opening and closing filtrations and their relevant persistence diagrams reveal information about spatial sizes of C1–C5 and H1–H4.

Figure 3e–j depicts an opening filtration of *f*. Opening operations with increasing structuring elements shrink the white regions. Thus, an opening filtration is good for discriminating sizes of holes. $X^{{\mathscr {O}}}_0=X_0$ is the smallest set in the opening filtration. We observe that as *i* increases, holes disappears. For instance, in Fig. 3g, the hole H1 disappears; in Fig. 3i, the hole H3 disappears; in Fig. 3h, the hole H2 disappears. This behavior can also be observed in the corresponding 1st level persistence diagram shown in Fig. 3q. Since $X^{{\mathscr {O}}}_0=X_0$, the holes H1, H2, and H3 are born at 0. Since H1 disappears in Fig. 3g, H1 has death value 2. Similarly, H3 has death value 3 and H2 has death value 2. Therefore, the death values of ${\mathscr {P}}_1(0)$ reveal sizes of 1D holes. Also, by (8), we know that $\beta _1(X^{{\mathscr {O}}}_0) = \# {\mathscr {P}}_1(0)$. This means that given a binary image *f*, or its black set $X_0$, individual sizes of $\beta _1(X_0)$ can be seen through ${\mathscr {P}}_1(0)$, where ${\mathscr {P}}_1$ is the 1-st level persistence diagram for the opening filtration of *f*.

Figure 3k–p depicts a closing filtration of *f*. Closing operations shrink the black regions. Thus, a closing filtration is good for discriminating sizes of connected components. In this case, $X^{{\mathscr {C}}}_0=X_0$ is the largest black set in the closing filtration: $X_{-5}^{{\mathscr {C}}} \subseteq \cdots \subseteq X_{-1}^{{\mathscr {C}}} \subseteq X_{0}^{{\mathscr {C}}}$. We define $Y_i = X_{-(5-i)}^{{\mathscr {C}}}$ for $i = 0,1, ..., 5$ for notational purposes and rewrite the closing filtration as $Y_0 \subseteq \cdots \subseteq Y_5$. Since $Y_5=X_0$ is the largest black set, as $Y_i$ decreases, connected components disappear. For instance, starting from Fig. 3p, connect component C3 disappears in $Y_2$; C2 disappears in $Y_1$; C1 disappears in $Y_0$. Since the ordering is reversed, C1 has birth value 0, C2 has birth value 1, and C3 has birth value 3. They each have the same death value of 5. Therefore, $\{ (b,5) \in {\mathscr {P}}_0(\{Y_i\})$ reveals sizes of connected components. This means that given a binary image *f*, or its black set $X_0$, spatial size information for connected components contributing to $\beta _0(X_0)$ can be seen through $\{ (b,5) \in {\mathscr {P}}_0(\{Y_i\})$, where ${\mathscr {P}}_0$ is the 0-th level persistence diagram for the considered closing filtration of *f*.

Figure 3q,r also reveal scale information about features. For example, the vertical gap between the points labeled H4 and H3 in Fig. 3q results from the separation in scale between the H4 feature and the H3 feature (the big “O” and the smaller scale white dot respectively in Fig. 3c). Similarly, the horizontal gap between the points labeled C4 and C1 in Fig. 3r corresponds to the separation in scale between the black regions with those labels in Fig. 3d. In this case, targeting the removal of features separated from H4 and C4 (and the largest feature C5), would result in an image with only the topological features shown in the original image Fig. 3a.

We now return to the more general erosion/dilation and opening/closing one-parameter filtrations presented earlier and extend these to form filtrations on grayscale images. Combining either of these filtrations with the sublevel set filtration in (9), one may obtain a *two-parameter filtration, or bi-filtration*. We take the opening filtration as an illustration. Given a grayscale image *g*, by (9), we have $g^{-1}_{t_i}(0) \subseteq g^{-1}_{t_j}(0)$ for any $t_i \le t_j$. Since for each *t*, $g_t$ is a binary image, by (13) we have that $O_{B_i}(g_t)^{-1}(0) \subseteq O_{B_j}(g_t)^{-1}(0)$. By combining both (9) and (13) we obtain18

This is a 2-filtration, an example of a multifiltration defined in Definition 5. In the next section, we formalize and extend the class of multifiltrations constructed from opening and closing operations on binary images and opening, closing, and thresholding operations for grayscale images.

## Multi-parameter filtrations

At this point, we have seen that erosion, dilation, opening, and closing each form one-parameter filtrations for binary images and that combining one of these operations with thresholding forms a 2-filtration (see Definition 5 below). As we show in the next example, opening and closing operations may also be combined to form a 2-filtration for a binary image. In fact, this process may be continued to define *k*-parameter filtrations, the overall goal of this section.

### Definition 5

(^23^) For $k \in {{\mathbb {N}}}$ and ${\mathbf {u}},~{\mathbf {v}}\in {\mathbb {Z}}^k$ we say that ${\mathbf {u}} \le {\mathbf {v}}$ if and only if $u_i \le v_i$ for all *i*. Given this partial order on ${\mathbb {Z}}^k$, a family of sets $\{S_{{\mathbf {i}}}\}_{{{\mathbf {i}}}\in A}$ with indexing set $A \subseteq {\mathbb {Z}}^k$ is a *multifiltration* (or *k*-*parameter filtration*) if for any ${\mathbf {u}}, {\mathbf {v}}\in A$ with ${\mathbf {u}}\le {\mathbf {v}}$, $S_{{\mathbf {u}}} \subseteq S_{{\mathbf {v}}}$.

Combining an opening filtration and a closing filtration and invoking Proposition 1 yields the following 2-filtration.19

**Figure 4:**
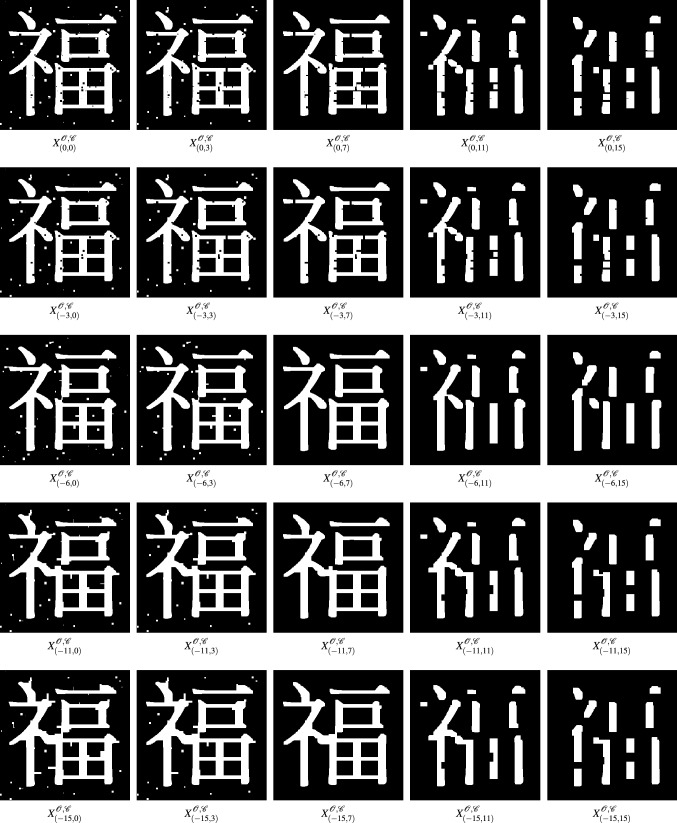
A 2-parameter filtration using structuring elements $B_i$ defined in (10). The notation $X^{{\mathscr {O}},{\mathscr {C}}}_{(-i,j)}$ denotes the set of black pixels in the image $(C_{B_i} \circ O_{B_j})(f)$ where *f* is the left-top image in the 2-parameter filtration. The formal definition can be found in Definition 6.

**Figure 5:**
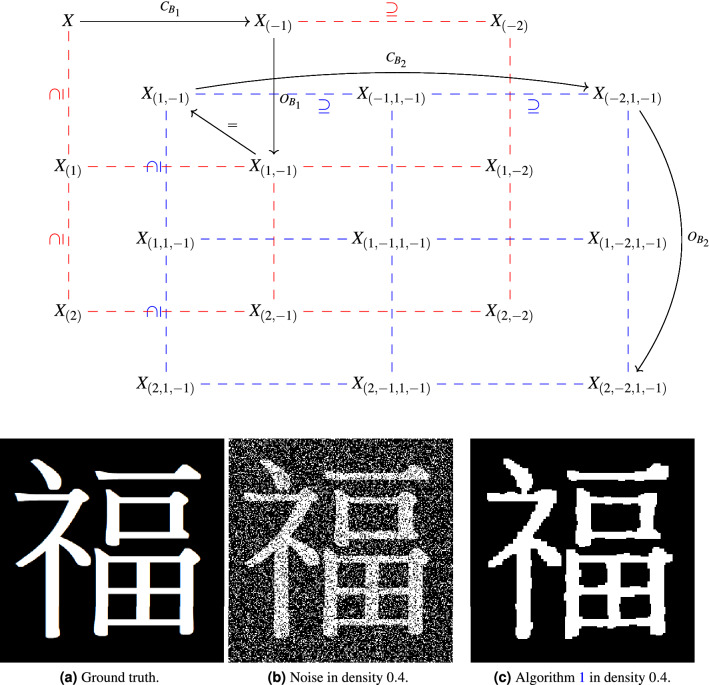
Top panel: Schematic illustration of steps in Algorithm 1 in the multiparameter filtration that would produce the alternating sequence $(2, -2, 1, -1)$. Red dotted lines highlight a bifiltration layer, and blue dotted lines highlight a different bifiltration layer. The black solid line represents the path and selections made by Algorithm 1. Bottom panel: An application. (**a**) Ground truth binary image. (**b**) Ground truth with salt and pepper noise with noisy density 0.4. (**c**) Denoised image produced by Algorithm 1. The parameters used for Algorithm 1 are MaxIter=10 and Sizetol=5. The resulting alternating opening/closing sequences ${{\mathbf {u}}}$ of (**c**) is $(-2,4,-1,3,-1,2,-1,1,-1)$.

**Figure 6:**
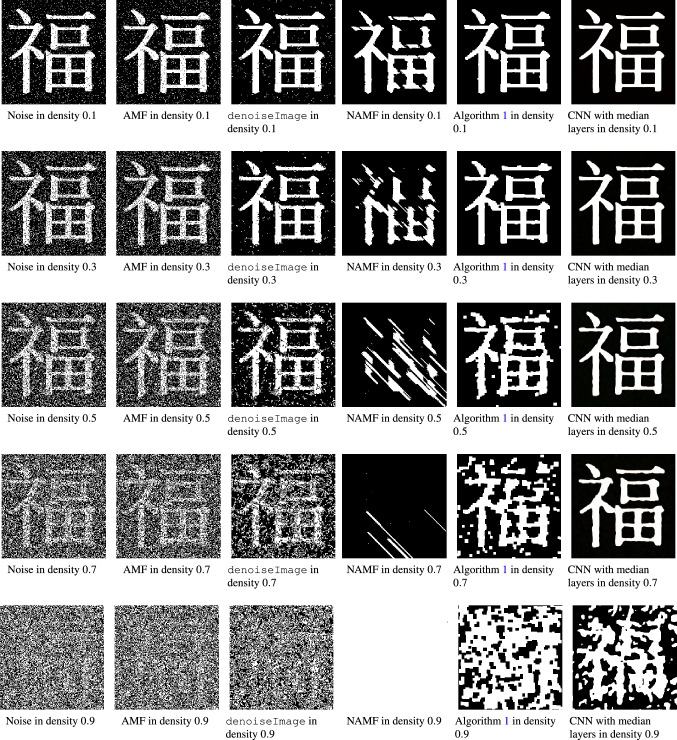
Conceptual images with salt and pepper noise and the results of different denoised algorithms: AMF^29^, denoiseImage, NAMF^31^, Algorithm 1, and CNN with median layers^30^. The parameters for Algorithm 1 are MaxIter=10 and Sizetol=5. Figure 5a is the ground truth image of the experiment.

**Figure 7:**
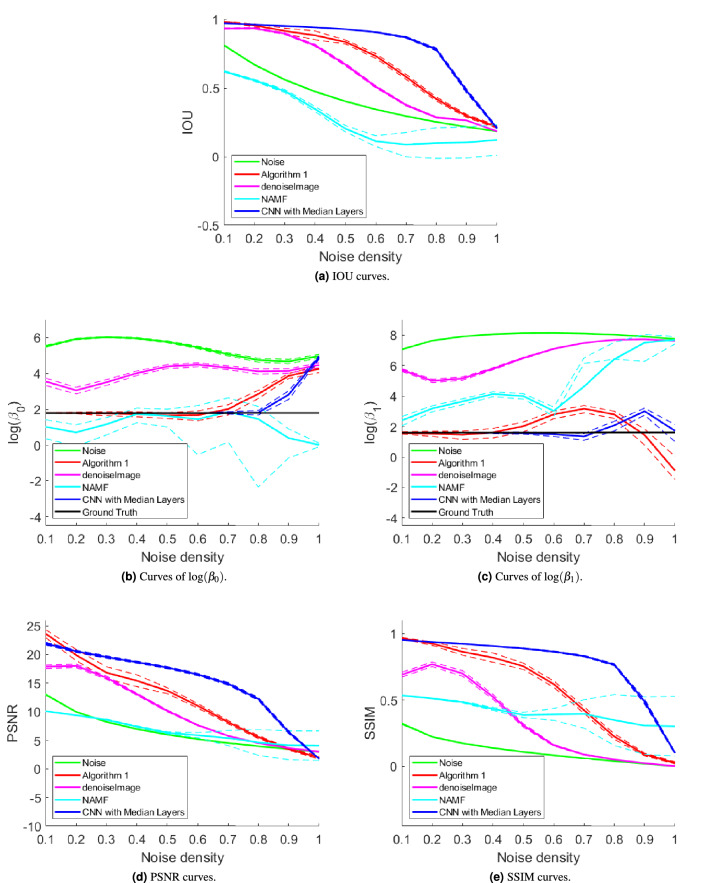
Mean (solid) plus standard deviation (dashed) curves of IOU, $\log (\beta _0)$, $\log (\beta _1)$, PSNR, and SSIM scores for 100 trials at each computed salt and pepper noise density. Images in Fig. 6 are the representatives of images for obtaining the results. See also Supplementary Table S1.

**Figure 8:**
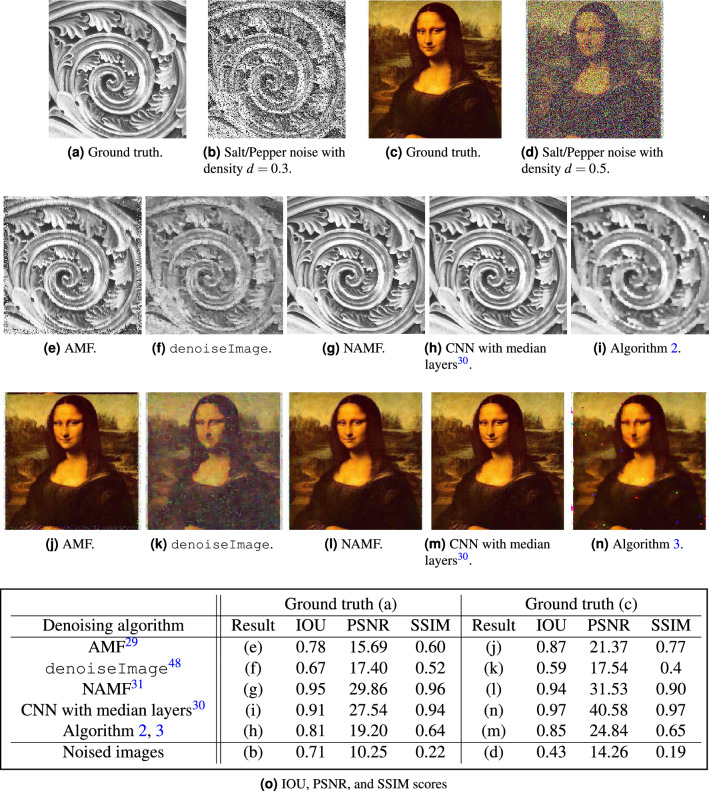
The ground truth images are (**a**), (**c**) where (**a**) is one of images from the open domain database Flickr Material Database^70^ and the full database can be accessed at https://people.csail.mit.edu/celiu/CVPR2010/FMD/. (**c**) Adopted from the Wikipedia page https://en.wikipedia.org/wiki/File:Mona_Lisa.jpg. The unprocessed images with noise are (**b**) and (**d**). The first two rows are the denoised images obtained by the following algorithms: AMF^29^, denoiseImage, NAMF^31^, Algorithm 2, 3, and CNN with median layers^30^. The last row is the averaged IOU, PSNR, and SSIM scores for the noised grayscale image for (**a**) and by color channel for (**c**).

**Figure 9:**
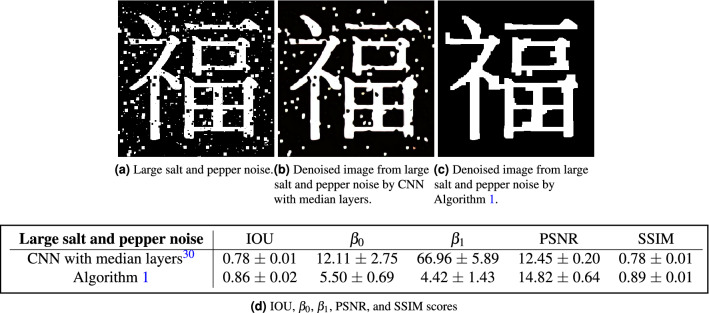
First row: (**a**) An example of image with larger spatial scale salt and pepper noise. (**b**) Corresponding denoised images produced by CNN with Median Layers. (**c**) Corresponding denoised images produced by Algorithm 1. Second row: The table of evaluation scores IOU, $\beta _0$, $\beta _1$, PSNR, and SSIM of CNN with median layers and Algorithm 1. For each noise type, 100 images were formed and tested. All scores are recorded by mean ± standard deviation for the 100 trials. The ground truth of the pair $(\beta _0, \beta _1)$ is (6, 5).

Figure 4 shows sample images from this opening/closing bifiltration as applied to an example of a kanji with (salt) noise. By visual inspection of the bifiltration depicted in Fig. 4, $X^{{\mathscr {O}},{\mathscr {C}}}_{(-6,7)}$ appears to be the most accurate rendering of the underlying kanji image. Since $X^{{\mathscr {O}},{\mathscr {C}}}_{(0,0)} \not \subseteq X^{{\mathscr {O}},{\mathscr {C}}}_{(-6,7)}$, there is no way to compare $X^{{\mathscr {O}},{\mathscr {C}}}_{(-6,7)}$ directly to the original image $X^{{\mathscr {O}},{\mathscr {C}}}_{(0,0)}$ using a one-filtration. However, multiple one-filtrations within the 2-filtration may be used to “connect” the two sets. Figure 3 shows the one-parameter persistent homology of opening and closing filtrations that can detect the holes and connected components in an image. However, the rows and columns in Figure 4 depict the underlying structure in single parameter opening/closing filtration would be influenced by original black/white components. This example illustrates that the 2-filtration build by opening and closing operations can keep the underlying structure of the image than one-parameter filtration via opening or closing operations. We discuss an approach for using persistent homology information to search for optimal renderings within multifiltrations, along with extensions of multifiltrations from “One‑parameter filtrations and persistent homology” section for binary images to a larger multifiltration that handles grayscale images, in “Application: a denoising algorithm for salt and pepper noise” section.

We now present a general multiparameter persistence framework using morphological operations. Consider a sequence of operations ${\mathscr {E}}_i:{\mathscr {I}}_P \rightarrow {\mathscr {I}}_P$ (e.g. erosion) and a sequence of operations, ${\mathscr {D}}_i:{\mathscr {I}}_P \rightarrow {\mathscr {I}}_P$ (e.g. dilation) satisfying the following: for any $f,~g\in {\mathscr {I}}_P$ and $i,j\in \{0, 1,2,\dots ,n\}$,

1. if $f \le g$, then ${\mathscr {E}}_i(f) \le {\mathscr {E}}_i(g)$ and ${\mathscr {D}}_i(f) \le {\mathscr {D}}_i(g)$;
2. if $i \le j$, then ${\mathscr {E}}_i(g) \ge {\mathscr {E}}_j(g)$ and ${\mathscr {D}}_i(g) \le {\mathscr {D}}_j(g)$;
3. $${\mathscr {D}}_0(g) = {\mathscr {E}}_0(g) = g$$.

When ${\mathscr {E}}_i= O_{B_i}$ and ${\mathscr {D}}_i= C_{B_i}$, assumptions (A1), (A2), and (A3) are similar to the *sieving axioms* in granulometry: anti-extensivity, increasingness, and the absorption property (^13,15^). Assumption (A1) is the *increasing property* seen also in Proposition 1. Assumption (A2) is the *absorption property* (^14^ Sec. 1.2.6, p.20). Finally, combining assumptions (A2) and (A3) would lead to the anti-extensive or extensive property.

In what follows, let ${\mathscr {E}}_i$ and ${\mathscr {D}}_i$, $i\in \{0, 1,2,\dots ,n\}$ be sequences satisfying (A1), (A2), and (A3). Consider $i\in \{0,\pm 1, ...,\pm n \}$ and define a function $M^{{\mathscr {E}}, {\mathscr {D}}}_i : {\mathscr {I}}_P \rightarrow {\mathscr {I}}_P$ as

20 $$\begin{aligned} M_i^{{\mathscr {E}}, {\mathscr {D}}}(g) := \left\{ \begin{array}{ll} {\mathscr {E}}_i(g) &{} \text{ for } i \ge 0\\ {\mathscr {D}}_{|i|}(g) &{} \text{ for } i<0 \end{array}\right. , \end{aligned}$$

and denote the 0-level set by

21 $$\begin{aligned} X_i^{{\mathscr {E}}, {\mathscr {D}}}(g) := \{ {{\mathbf {x}}}\in P \ | \ M_i^{{\mathscr {E}}, {\mathscr {D}}}(g)({{\mathbf {x}}})=0 \} = M_i^{{\mathscr {E}}, {\mathscr {D}}}(g)^{-1}(0). \end{aligned}$$

When the context is understood, we sometimes abbreviate $M^{{\mathscr {E}}, {\mathscr {D}}}_i$ as $M_i$, and $X^{{\mathscr {E}}, {\mathscr {D}}}_i(g)$ as $X_i$. The notation (20) unifies the operations ${\mathscr {E}}$ and ${\mathscr {D}}$ in the following way.

### Lemma 1

Let $i,j \in \{0,\pm 1,\ldots ,\pm n \}$ and $g\in {\mathscr {I}}_P$ be a binary image. Suppose ${\mathscr {E}}_i$ and ${\mathscr {D}}_i$ satisfy (A1), (A2), and (A3). If $i \le j$, then $M_j^{{\mathscr {E}}, {\mathscr {D}}}(g) \le M_i^{{\mathscr {E}}, {\mathscr {D}}}(g)$.

### Proof

Let $i\le j$. Suppose first that $i\ge 0$. Then $M_j^{{\mathscr {E}}, {\mathscr {D}}}(g) = {\mathscr {E}}_j(g) \le {\mathscr {E}}_i(g) = M_i^{{\mathscr {E}}, {\mathscr {D}}}(g)$. If, on the other hand, $i < 0$, then there are two cases. In the case when $j > 0$, then $M_j^{{\mathscr {E}}, {\mathscr {D}}}(g) = {\mathscr {E}}_j(g) \le g \le {\mathscr {D}}_{|i|}(g) = M_i^{{\mathscr {E}}, {\mathscr {D}}}(g)$. In the second case where $j\le 0$, then since $i \le j \le 0$, $|j| \le |i|$ and we obtain $M_j^{{\mathscr {E}}, {\mathscr {D}}}(g) = {\mathscr {D}}_{|j|}(g) \le {\mathscr {D}}_{|i|}(g) = M_i^{{\mathscr {E}}, {\mathscr {D}}}(g)$. $\square$

The essential step in obtaining a multi-parameter filtration is to apply $M^{{\mathscr {E}},{\mathscr {D}}}$ inductively. This requires us to extend the notation of (20) and (21) to a multi-index ${\mathbf {i}}\in {\mathbb {Z}}^k$.

### Definition 6

Let $\{{\mathscr {E}}_i\}$ and $\{{\mathscr {D}}_i\}$ be sequences of morphological operations that satisfy (A1), (A2), and (A3). For $k, n \in {\mathbb {N}}$ and ${\mathbf {i}} = (i_1, i_2, \dots , i_k) \in \{0, \pm 1,..., \pm n \}^k$, we define $M^{{\mathscr {E}},{\mathscr {D}}}_{{\mathbf {i}}}:{\mathscr {I}}_P \rightarrow {\mathscr {I}}_P$ and $X^{{\mathscr {E}},{\mathscr {D}}}_{{\mathbf {i}}}\subseteq P$ by

22 $$\begin{aligned} M^{{\mathscr {E}},{\mathscr {D}}}_{{\mathbf {i}}}(g) = (M^{{\mathscr {E}},{\mathscr {D}}}_{i_1} \circ M^{{\mathscr {E}},{\mathscr {D}}}_{i_2} \circ \cdots \circ M^{{\mathscr {E}},{\mathscr {D}}}_{i_k})(g), \end{aligned}$$

and

23 $$\begin{aligned} X^{{\mathscr {E}},{\mathscr {D}}}_{{\mathbf {i}}}(g) = \{ {{\mathbf {x}}}\in P \ | \ M^{{\mathscr {E}},{\mathscr {D}}}_{{\mathbf {i}}}(g)({{\mathbf {x}}}) = 0 \} = M^{{\mathscr {E}},{\mathscr {D}}}_{{\mathbf {i}}}(g)^{-1}(0). \end{aligned}$$

Similarly, we abbreviate the notation $M^{{\mathscr {E}},{\mathscr {D}}}_{{\mathbf {i}}}$ as $M_{{\mathbf {i}}}$ and $X^{{\mathscr {E}},{\mathscr {D}}}_{{\mathbf {i}}}(g)$ as $X_{{\mathbf {i}}}$ if operations ${\mathscr {E}}, {\mathscr {D}}$ and image *g* are specified.

For example, for ${{\mathbf {i}}}=(-1,1)$, $M_{{\mathbf {i}}}(g) = M_{-1}(M_1(g))$ means that the image *g* is filtered by ${\mathscr {E}}_1$ followed by ${\mathscr {D}}_1$, i.e. $M_{{\mathbf {i}}}(g) = M_{-1} \circ M_1(g) = {\mathscr {E}}_1 ({\mathscr {D}}_1(g) )$.

Motivated by (19), we consider the sets $X_{{\mathbf {i}}}$ formed by the application of alternating ${\mathscr {E}}$ and ${\mathscr {D}}$ operations. Using (20), we see that alternating these operations corresponds to a multi-index consisting of an alternating sequence of integers.

### Definition 7

The sequence ${\mathbf {i}}=(i_1, i_2, \ldots , i_k)\in \{0, \pm 1,..., \pm n \}^k$ is an *alternating sequence* if $i_{l} \cdot i_{l+1} \le 0$ for all $l \in \{ 1,2, ..., k\}$. By extension, the set $A\subseteq \{0, \pm 1,..., \pm n \}^k$ is a *set of alternating sequences* if it contains only *alternating sequences*.

We are now ready to present our main theorem: alternating the operations ${\mathscr {E}}$ and ${\mathscr {D}}$ leads to a multi-parameter filtration.

### Theorem 1

Let *g* be a binary image, and $A \subseteq \{0,\pm 1, ...,\pm n \}^k$ be a set of alternating sequences. Assume ${\mathscr {E}}_i$, ${\mathscr {D}}_i: {\mathscr {I}}_P \rightarrow {\mathscr {I}}_P$, $i\in \{1,2,\dots ,n\}$ satisfy (A1), (A2) and (A3). Then $\left\{ X^{{\mathscr {E}}, {\mathscr {D}}}_{{\mathbf {i}}}(g) \right\} _{{\mathbf {i}}\in A}$ is a *k*-parameter filtration.

### Proof

Let $\mathbf{u } = (u_1, ..., u_n), \mathbf{v } = (v_1, ..., v_n) \in A$ and $\mathbf{u } \le \mathbf{v }$. By Definition 5, we need to verify that $M_{\mathbf{u }}(g)^{-1}(0) \subseteq M_{\mathbf{v }}(g)^{-1}(0)$.

By Lemma 1, since $u_n \le v_n$ we have that $M_{v_n}(g) \le M_{u_n}(g)$. Applying (A1), we see that

24 $$\begin{aligned} (M_{v_{n-1}} \circ M_{v_n})(g) \le (M_{v_{n-1}} \circ M_{u_n})(g). \end{aligned}$$

Since $u_{n-1} \le v_{n-1}$ by Lemma 1 again, we have

25 $$\begin{aligned} (M_{v_{n-1}} \circ M_{u_n})(g) \le (M_{u_{n-1}} \circ M_{u_n})(g). \end{aligned}$$

Therefore, by combining (24) and (25), we prove that $(M_{v_{n-1}} \circ M_{v_n})(g) \le (M_{u_{n-1}} \circ M_{u_n})(g)$. Finally, by applying the argument inductively one may conclude that

$$\begin{aligned} \begin{aligned} M_{\mathbf{v }}(g)&= (M_{v_1} \circ \cdots \circ M_{v_n})(g) \le (M_{u_1} \circ \cdots \circ M_{u_n})(g) = M_{\mathbf{u }}(g). \end{aligned} \end{aligned}$$

By Proposition 2, we conclude that $M_{\mathbf{u }}(g)^{-1}(0) \subseteq M_{\mathbf{v }}(g)^{-1}(0)$. $\square$

### Remark 4

For purposes of exposition and to align with common practices in using morphological operations in image smoothing, we focused the composition of operations on alternating sequences (see^13–15^). This is inherent in (A2) as well as the stipulation that the indexing set *A* in Theorem 1 consists of alternating sequences. We note here, however, that the theorem holds true even if *A* contains sequences that are not alternating.

We now discuss examples to illustrate the framework given in Theorem 1. As a first example, consider erosion and dilation given as ${\mathscr {E}}_i := \varepsilon _{B_i}$ and ${\mathscr {D}}_i := \delta _{B_i}$. For this pair of operations, (A1) follows from Proposition 1, (A2) follows from Proposition 3, and (A3) is clear.

Therefore, by Theorem 1, $\left\{ X^{\varepsilon , \delta }_{{\mathbf {i}}}\right\} _{{\mathbf {i}}\in A}$ forms a multi-parameter filtration, where *A* is any set of alternating sequences. As a second example, consider the opening and closing operations and let ${\mathscr {E}}_i := O_{B_i}$ and ${\mathscr {D}}_i := C_{B_i}$. By Theorem 1, $\left\{ X^{{\mathscr {O}},{\mathscr {C}}}_{{\mathbf {i}}}\right\} _{{\mathbf {i}}\in A}$ forms a multi-parameter filtration. In fact, erosion/closing, and opening/dilation would also lead to multiparameter filtrations. It is important to note that while erosion/dilation and opening/closing lead naturally to multiparameter filtrations, the top-hat transformations do not. As mentioned in “One‑parameter filtrations and persistent homology” section, these transformations do not satisfy (A1) and (A3) in general and, therefore, do not satisfy the hypotheses of Theorem 1.

At this point, *g* is assumed to be a binary image. If *g* is a grayscale image, one may combine the sublevel set filtration with the multiparameter filtration described in Theorem 1 to obtain another multiparameter filtration. In the rest of this section, we will formulate this concept as an extension of Theorem 1.

Let $\{ {\mathscr {E}}_i \}_{i = 1}^n$ and $\{ {\mathscr {D}}_i \}_{i = 1}^n$ be sequences of operations ${\mathscr {I}}_P \rightarrow {\mathscr {I}}_P$ satisfying (*A*1), (*A*2) and (*A*3). Moreover, we also require that for all $i \in \{ 1,2, ..., n\}$ and $t \in \{ 0,1,,2 ..., N \}$,

(A4) $${\mathscr {E}}_i \circ \tau _t = \tau _t \circ {\mathscr {E}}_i$$ and $${\mathscr {D}}_i \circ \tau _t = \tau _t \circ {\mathscr {D}}_i$$.

This assumption means that the morphological operations and thresholding operation commute. Proposition 4 shows that $\delta$, $\varepsilon$, *O*, *C* satisfy (A4).

For every ${{\mathbf {u}}}\in \{ 0, \pm 1, ..., \pm n\}^k$ let $X_{t,{\mathbf {u}}}^{{\mathscr {E}},{\mathscr {D}}} := M_{{\mathbf {u}}}(f)_t^{-1}(0).$ We now show that if (A1)-(A4) are satisfied, then $\{X_{t,{\mathbf {u}}}^{{\mathscr {E}},{\mathscr {D}}}\}_{(t, {\mathbf {u}})}$ forms a $(k+1)$-parameter filtration. To achieve that, we need to verify that $M_{{\mathbf {v}}}(f_s) \le M_{{\mathbf {u}}}(f_t)$, for all $(t,{\mathbf {u}}) \le (s, {\mathbf {v}})$. By (A4), we have $M_{{{\mathbf {u}}}}(f)_t = M_{{{\mathbf {u}}}}(f_t)$ for $t \in \{ 0,1,,2 ..., N \}$. Therefore, by (A4) and Theorem 1, one has

$$\begin{aligned} M_{{\mathbf {v}}}(f_s) \le M_{{\mathbf {u}}}(f_s) = M_{{\mathbf {u}}}(f)_s \le M_{{\mathbf {u}}}(f)_t = M_{{\mathbf {u}}}(f_t). \end{aligned}$$

We summarize the above discussion into the following result.

### Corollary 1

Let *g* be a grayscale image, and $A \subseteq \{0,\pm 1, ...,\pm n \}^k$ be a set of alternating sequences. Assume ${\mathscr {E}}_i$, ${\mathscr {D}}_i : {\mathscr {I}}_P \rightarrow {\mathscr {I}}_P$, $i\in \{1,2,\dots ,n\}$ satisfy (A1), (A2), (A3), and (A4). Then $\{X^{{\mathscr {E}},{\mathscr {D}}}_{t,{\mathbf {u}}}(g) \}_{(t,{\mathbf {u}})}$ is a $(k+1)$-parameter filtration.

The following is an example of the framework in Theorem 1,26While different methods, including the rank invariant function^24^ and sheaf theory^46,47^, have been developed to study multi-parameter persistence, for purposes of illustration we will focus on computing persistent homology along *nondecreasing paths* in the constructed multifiltration.

### Definition 8

Define a *nondecreasing path* in indexing set *A* as a sequence ${\mathbf {u}}_0, {\mathbf {u}}_1, \ldots , {\mathbf {u}}_l \in A$ such that ${\mathbf {u}}_i \le {\mathbf {u}}_{i+1}$ for all $i=0,\ldots , l$. Then for a multifiltration $\{X_{{\mathbf {u}}}\}_{{\mathbf {u}}\in A}$ and nondecreasing path ${\mathbf {u}}_0, {\mathbf {u}}_1, \ldots , {\mathbf {u}}_l$ in *A*, $\{X_{{\mathbf {u}}_i}\}_{i}$ is a one-parameter filtration.

As we outline in the following section, this structure allows us to systematically extract information about geometric scale and optimize for certain topological features. Following multiple or successive nondecreasing paths allows for greater exploration of the multifiltration.

## Application: a denoising algorithm for salt and pepper noise

In this section, we use a multiparameter filtration to construct a denoising algorithm aimed at removing salt and pepper noise. In particular, one goal of the proposed algorithm is removing small scale white/black regions from the images in order to focus on the larger scale features as shown in Fig. 3 where C1–C3 and H1–H3 are of small scale relative to C4–C5 and H4. We note that the proposed multiparameter framework may be extremely large/high dimensional and contain a lot of useful information about the image and noise. We propose a denoising algorithm in this section to demonstrate one way to extract some useful information from the multiparameter filtration. Investigating additional methods for extracting information from large multiparameter filtrations in order to more fully exploit the information they contain is an interesting topic for the future work.

The outline of this section is as follows. We first describe the denoising algorithm which is designed for binary images (“Denoising algorithm for binary images” section). Some state-of-art denoised algorithms are also considered in the experiment, such as the denoiseImage function in Matlab^48^, *adaptive median filter* (AMF)^29^, *non-local adaptive mean filter* (NAMF)^31^, and a *convolutional neural network* (CNN) with median layers^30^. These algorithms and evaluation metrics are introduced in “Other methods and evaluation metrics” section. To measure its performance, we demonstrate it on synthetic images (“Experiments with salt and pepper noise” section) and extend the algorithm to grayscale images, and RGB color images (“Extensions to grayscale and RGB images” section). Throughout, we compare the results of this algorithm (Algorithm 1) to those obtained by previous methods. Lastly, to show the difference between Algorithm 1 and machine learning models, we compare the performance of Algorithm 1 and the CNN with median layers on image with big salt and pepper noise.

### Denoising algorithm for binary images

Given a binary image *f*, we wish to apply alternating opening/closing operations to *f*. Traditionally, this requires visual inspection to tune the size of the utilized structuring elements as well as the number of operations performed. We now seek to automate this process by more fully utilizing the full multiparameter persistence framework.

The proposed algorithm is iterative. In each iteration, we will use persistence diagrams computed along a nondecreasing path in the multiparameter filtration to guide the choice of a structuring element used for opening or closing. To get started, consider a binary image *f* contaminated by salt and pepper noise. That is, certain pixels have been switched to either white (salt) or black (pepper). To simulate salt and pepper noise, we use the Matlab function imnoise with *noise density* as a parameter to determine the density or proportion of contaminated pixels. For example, if the density equals 0.1, the imnoise function randomly changes $10\%$ pixels in the image domain to be black or white. See e.g. the first column of Fig. 6 for examples of images contaminated by the salt and pepper noise with different noise densities.

We first consider the closing filtration of *f*, $\{Y_j\}_{j=0}^n$, where $Y_j = X^{{\mathscr {C}}}_{n-j}$ for $j = 0,1, ..., n$. By (14), we have a filtration $Y_0 \subseteq Y_1 \subseteq Y_2 \subseteq \cdots \subseteq Y_n$ of $(n+1)$ sets and the 0-th persistence diagram ${\mathscr {P}}_0^{{\mathscr {C}}} := {\mathscr {P}}_0[\{ Y_j \}_{j = 0}^n]$ (cf. Figure 3(r)). Similar to the discussion in “New filtrations based on morphological operations” section, the persistence diagram ${\mathscr {P}}_0^{{\mathscr {C}}}$ contains features (connected black regions) that are present in the original image $X^{{\mathscr {C}}}_0=f^{-1}(0)$, and births *b* in ${\mathscr {P}}_0^{{\mathscr {C}}}$ indicate the size of the corresponding feature by giving the amount of closing required to remove it from the image. Since salt and pepper noise creates features that are small in spatial scale, we take a conservative route by choosing a level of minimal level of closing that removes at least one, smallest spatial scale feature using

$$\begin{aligned} i_c = (n + 1) - \max \{b : (b,n+1) \in {\mathscr {P}}_0^{{\mathscr {C}}} \}. \end{aligned}$$

The chosen binary image after the first step is then $X_{i_c}:=C_{B_{i_c}}(f)^{-1}(0)$. To generalize this approach, we note that a gap in the death coordinate values in ${\mathscr {P}}_0^{{\mathscr {C}}}$ can be used to detect a separation in spatial scales for features in the original image. The structuring element index $i_c$ could then be chosen to target the beginning of this gap.

At this point $i_c$ is chosen and is fixed. Using the new binary image $C_{B_{i_c}}(f)$, we now consider the opening filtration of $C_{B_{i_c}}(f)$, $\{X^{{\mathscr {O}}}_i\}_{i=0}^n$, as shown in (13), where in this case $X^{{\mathscr {O}}}_i = O_{B_{i}}(C_{B_{i_c}}(f))^{-1}(0)$. As discussed in “New filtrations based on morphological operations” section, ${\mathscr {P}}_1^{{\mathscr {O}}} := {\mathscr {P}}_1(\{X^{{\mathscr {O}}}_i\}_{i=0}^n)$ reveals size information of the white regions. As demonstrated in “One‑parameter filtrations and persistent homology” section, ${\mathscr {P}}_1^{{\mathscr {O}}}$ contains features that are present in the original binary image, $X^{{\mathscr {O}}}_0$, and $d\in {\mathscr {P}}_1^{{\mathscr {O}}}$ indicates the spatial size of the feature, that is, the amount of opening required to remove the feature. Similar to our approach in the first step using closing, we now choose the size of the structuring element for opening to be

$$\begin{aligned} i_o = \min \{d : (0,d) \in {\mathscr {P}}_1^{{\mathscr {O}}} \}. \end{aligned}$$

The binary image following the second step is now $X_{(i_o, -i_c)} = O_{B_{i_o}}(C_{B_{i_c}}(f))^{-1}(0)$.

We repeat this alternating process. The stopping criterion is when the selected structuring element size exceeds a preset maximum, $\texttt {SizeTol}$. This could be given by the size of the image, or given as an upper bound on the spatial size of noisy features or features we wish to remove. The algorithm is summarized in Algorithm 1. Figure 5 illustrates Algorithm 1 in a schematic way in the multiparameter space.

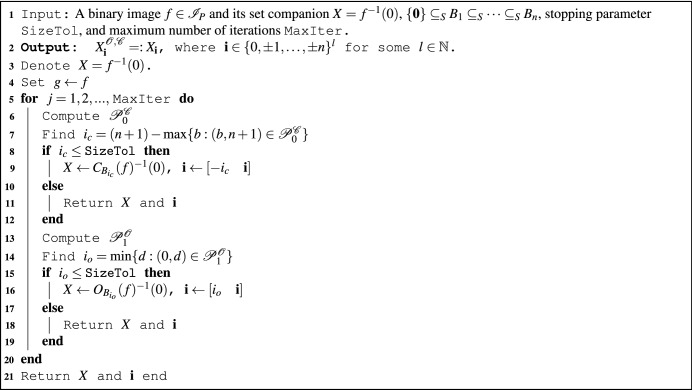



To demonstrate Algorithm 1, we use the $190 \times 190$ binary image shown in the bottom panel of Fig. 5a as the ground truth. We add salt and pepper noise with noise density 0.4 to the ground truth and the contaminated image is shown in Fig. 5b. The denoised image produced by Algorithm 1 can be found in Fig. 5c. The alternating sequence obtained by Algorithm 1 is $(-2,4,-1,3,-1,2,-1,1,-1)$, which means that the denoised image in Figure 5(c) is obtained by alternating opening and closing operations in the following order: $C_{B_2}$, $O_{B_4}$, $C_{B_1}$, $O_{B_3}$, $\cdots$, $C_{B_1}$. We also observe that Algorithm 1 is unsupervised, meaning that it does not involve any training process. It solely uses information from the persistence diagrams as well as the preset upper bound on the utilized structuring elements. Visually, the denoised image produced by Algorithm 1 is close to the ground truth. We now turn our focus to testing Algorithm 1 in a variety of settings, and gauging its performance through comparison with existing methods.

### Other methods and evaluation metrics

To compare, we consider the following algorithms in the literature: the denoiseImage function provided by Matlab 2019, *adaptive median filter* (AMF)^29^, *non-local adaptive mean filter* (NAMF)^31^, and a *convolutional neural network* (CNN) with median layers^30^. The denoiseImage function is deep learning based^48^. AMF is a typical method for erasing salt and pepper noise by computing median pixels values of local image regions. The NAMF algorithm uses non-local means (NLM)^31^ to detect and estimate the locations and strengths of salt and pepper noise features. The CNN with median layers integrates median layers into the convolutional neural network for removing salt and pepper noise and is considered to be a state-of-art method for removing salt and pepper noise. Note that among these methods, AMF and NAMF are unsupervised while denoiseImage and CNN with median layers are supervised (deep learning based).

In this work, for the denoiseImage, AMF, NAMF, and CNN with median layers method, we use each code’s default parameters, where only Algorithm 1 and NAMF provide manual parameters. For each prescribed noise density, 100 images were formed, Algorithm 1 with MaxIter=10 and SizeTol=5 was applied to each. The parameters *Ds*, *ds* and *B* of the NAMF algorithm were 2, 20,  and 0.8 suggested in the demo code that was released by the authors. The algorithm denoiseImage is a built-in Matlab function for denoising the image by using the deep neural network trained on the dataset provided by Zhang *et al.*^48^. On the other hand, Liang *et al.* generated the dataset of images with salt/pepper noises for training the CNN with median layers method^30^.

We now introduce metrics to further measure the performances of these denoising algorithms. First, we consider the Betti numbers: $\beta _0$ and $\beta _1$. As shown in Fig. 5a, the Betti numbers for the ground truth are $(\beta _0, \beta _1) = (6,5)$. In addition, we consider some more standard metrics: the *intersection over union* (IOU)^49^, *peak signal-to-noise ratio* (PSNR)^50,51^, and *structural similarity* (SSIM)^52^. We use $\texttt {jaccard}$, ${\texttt {psnr}}$, and ${\texttt {ssim}}$ in Matlab to compute IOU, PSNR, and SSIM, respectively. For reference, we include the definitions of these metrics below. Let *f* and ${\widehat{f}}$ be binary images. The IOU score of binary images $f, {\widehat{f}}$ is defined as

$$\begin{aligned} \mathrm{IOU}(f, {\widehat{f}}) = \frac{| f^{-1}(0) \cap {\widehat{f}}^{-1}(0) | }{| f^{-1}(0) \cup {\widehat{f}}^{-1}(0) |}. \end{aligned}$$

If the IOU score is 1, this means that two binary images *f* and ${\widehat{f}}$ are identical, so the higher the IOU, the better. The PSNR score is defined as

$$\begin{aligned} \mathrm{PSNR}(f, {\widehat{f}}) = 10 \cdot \log _{10} \left( \frac{ \left( \max _{{{\mathbf {x}}}\in P} f({{\mathbf {x}}}) \right) ^2}{\mathrm{MSE}(f, {\widehat{f}})} \right) , \text { where }{\mathrm{MSE}(f, {\widehat{f}})} = \frac{1}{|P|} \sum _{{{\mathbf {x}}}\in P} (f({{\mathbf {x}}}) - {\widehat{f}}({{\mathbf {x}}}))^2. \end{aligned}$$

Note that the IOU score is defined for the binary images while the PSNR can be used for grayscale images. The higher the PSNR, the better. Generally speaking, when the PSNR is greater than 30, it is difficult for human eyes to distinguish the difference between the two images^53^. Lastly, the SSIM score of $(f, {\widehat{f}})$ based on various windows of images. The measure between two windows (subimages) *W* and *V* is defined as

$$\begin{aligned} {\text {SSIM(W,V)}} = \frac{(2 \mu _{W}\mu _{V} + C_1) (2 \sigma _{WV} + C_2) }{(\mu _{{W}}^2 + \mu _{{V}}^2 + C_1)(\sigma _{{W}}^2 + \sigma _{{V}}^2 + C_2) }, \end{aligned}$$

where $\mu _W$ represents the average of *W*, $\sigma _{{W}}^2$ represents the variance of *W*, and $\sigma _{WV}$ represents the covariance of *W* and *V*. For the typical choice of *W*, *V*, $C_1$, and $C_2$, we refer readers to^52^. As mentioned above, we use Matlab built-in function $\texttt {ssim}$ with default parameters. Similar to other scores, a higher SSIM score indicates that two images have a higher degree of similarity. If SSIM score of $(f, {\widehat{f}})$ is 1, this means that two images are identical. The SSIM and PSNR scores are typical metrics used in image denoising tasks^30,31^. On the other hand, in the image segmentation tasks^54,55^, the IOU score (or the Jaccard index^49^) measures the distances between two binary masks of images.

### Experiments with salt and pepper noise

To further test Algorithm 1, we again use the $190 \times 190$ binary image shown in Fig. 5a as the ground truth. We add salt and pepper noise to the ground truth with various levels of noise densities. Specifically, we use the Matlab built-in function imnoise along with a specified noise density parameter *d* with range $\{ 0.1, 0.2,\ldots , 1.0 \}$. As illustration, resulting contaminated images with noise density 0.1, 0.3, 0.5, 0.7, and 0.9 can be found in the first column of Fig. 6. For each noise density, we construct 100 contaminated images with the prescribed noise density. We then apply various denoising algorithms on each collection of contaminated images and measure their accuracy by comparing results to the ground truth.

Figure 6 demonstrates sample contaminated images with different noise densities as well as the processed images produced by the various denoising methods. From first to seventh column of Fig. 6, they are contaminated images, denoised images by AMF, denoiseImage, NAMF, Algorithm 1, and CNN with median layers, respectively. Visually, the denoised images produced by CNN with median layers (7th column of Fig. 6) are the closest to the ground truth. Compared with the unsupervised methods (i.e., AMF and NAMF), denoised images by Algorithm 1 are closest to the ground truth. Even in the case with high noise density 0.7, the denoised image returned by Algorithm 1 still recovers much of the core structure of the ground truth Fig. 5a. On the other hand, denoised images by AMF (2nd column of Fig. 6) and denoiseImage (3rd column of Fig. 6) still appear pixelated. We also see that although salt and pepper features seem to be removed by NAMF (4th column of Fig. 6), features from the ground truth image are also changed drastically.

Figure 7 shows results for studies of 100 noisy images with the prescribed noise densities. Since the AMF algorithm requires more computing costs ($\approx 2$ minutes per $190 \times 190$ binary image) and NAMF has been shown to perform better among principal non-learning based methods^31^, we omit AMF from this experiment. General speaking, in Fig. 7, one may consider the curves labeled as “Noise” in each subplots as the baseline or the worst case scenario. Figure 7a shows a plot of the resulting IOU scores. We observe that in Fig. 7a, CNN with median layers performs the best followed by our proposed Algorithm 1. Figure 7b,c shows the log plot of Betti numbers. The ground truth of Betti numbers (6, 5) are labeled as “Ground Truth” in both Fig. 7b,c. In other words, in (b)-(c), curves that are close to the horizontal line “Ground Truth” are better. We observe that in Fig. 7b,c CNN with median layers and Algorithm 1 outperform the rest methods. Figure 7d shows the resulting values of PSNR. We observe in Fig. 7d that Algorithm 1 performs better than CNN with median layers at noise density 0.1, and better than $\texttt {denoiseImage}$ and NAMF. Figure 7e shows the resulting values of SSIM. In (e), we observe that NAMF seems to be unchanged as noise density increases. We again see that the performance of Algorithm 1 is in between that of CNN with median layers and $\texttt {denoiseImage}$. The numerical values computed to produce Fig. 7 can be found in Supplementary Table S1.

### Extensions to grayscale and RGB images

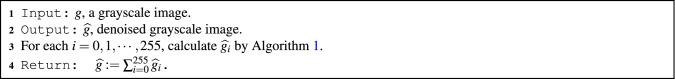

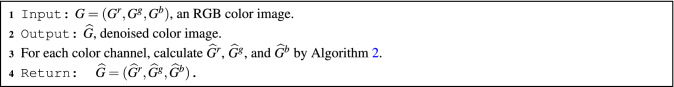





Combining Algorithm 1 with the thresholding techniques in Proposition 4 extends this approach to grayscale images. For a grayscale image $g : P \rightarrow \{ 0,1, ..., 255\}$, consider its binary images via global thresholding (6): $g_0, g_1,..., g_{255}$. We apply Algorithm 1 to each binary image $g_i$ and obtain a denoised binary image $\widehat{g_i}$. The final output grayscale image would be the sum of $\sum _{i=0}^{255}\widehat{g_i}$ as shown in Algorithm 2. We apply Algorithm 2 with the parameters $(\texttt {SizeTol}, \texttt {MaxIter})=(4,10)$ to a grayscale image as shown in Fig. 8b, and the denoised image is shown in Fig. 8i. We observe that in Fig. 8i, the salt and pepper noise is removed, and some portions of images (e.g. the mouth of the man, the camera) are blurry. Note that since each thresholded image, $g_i$, is treated separately by Algorithm 1, the resulting images $\widehat{g_i}$ may not form a filtration, i.e. $\widehat{g_i}^{-1}(0) \nsubseteq \widehat{g_j}^{-1}(0)$ for $i\le j$. It would be interesting to extend Algorithm 1 to an approach that would preserve the subset relations on the denoised images, ensuring that they form a filtration.

By comparison, the denoised images produced by AMF, denoiseImage, NAMF, Algorithm 2, and CNN with Median Layers are shown in the Fig. 8e–h, respectively. We observe that, visually, denoised images produced by NAMF, Algorithm 2, and CNN with Median Layers seem to perform the best, while the image produced by denoiseImage seems to be pixelated and the one produced by AMF seems to be problematic on the boundaries of the image. To quantify the performances, Fig. 8o shows the IOU, PSNR, and SSIM scores when comparing the ground truth image Fig. 8a with denoised images (e)–(i), respectively. In this case, the denoised image produced by NAMF as shown in Fig. 8g earns the best scores and the image produced by Algorithm 2 as shown in Fig. 8h earns one of the top 3 scores among these methods.

Using a similar procedure, we may also extend the Algorithm 1 approach to RGB color images. Here, we treat each of the three color channels as a grayscale image and follow the same procedure described for grayscale images above as shown in Algorithm 3. The results for the 3 channels are then viewed together as an RGB image. Figure 8d shows the constructed noisy image from the original RGB image Fig. 8c. Figure 8m is the image produced by Algorithm 3 with the parameter $(\texttt {SizeTol}, \texttt {MaxIter})=(7,10)$. We observe that almost all of the salt and pepper noise is removed, and moreover, the denoised image still preserves the original image well. As a comparison, the denoised images by AMF, denoiseImage, NAMF, Algorithm 3, and CNN with Median Layers are shown in the Fig. 8j–n, respectively. Their IOU, PSNR, and SSIM scores can be found in Fig. 8o, and we find that again Algorithm 3 performs in the top 3 among these methods.

Our focus in this section has been to demonstrate that the multiparameter filtration contains useful information and that automation may be used to extract it. Our proposed denoising algorithm works well on binary, grayscale, and color images with salt and pepper noise where the separation in spatial scale between the noise and true features may be used to effectively remove noise. Recently, others have developed salt and pepper denoising algorithms using deep learning e.g.^56,57^, where a training process is required. Our unsupervised approach does not require training and it would be interesting to investigate whether combining the two approaches could lead to even better results.

Hyper parameter tuning for the proposed method is also an important task. Currently, we choose the parameters based on the assumption that features due to noise have a relatively small spatial scale which we estimate based on empirical results. An interesting direction for future work is to more fully develop an automatic criterion for locating the gap in the persistence diagram that indicates a separation in spatial scales, using this information to set the appropriate hyper parameters.

### Experiments with larger spatial scale noise

At this point, we have seen that the proposed algorithms, Algorithm 1 and extensions Algorithm 2 and 3, are capable of processing and removing salt and pepper noise. They are unsupervised and use information from the proposed multi-parameter filtration. Their performances can be better than the general purpose deep learning based denoising algorithm, $\texttt {denoiseImage}$ (as shown in Fig. 7). The performance of CNN with Median Layers seems to be the best among methods we tested. The focus of this subsection is to conduct a new experiment to further compare Algorithm 1 and CNN with Median Layers.

As mentioned in “One‑parameter filtrations and persistent homology” section, persistence diagrams for opening/closing filtrations reveal spatial scale information about features. If there exists a gap in the persistence diagrams, it may suggest that features exist in at least two different spatial scales. To quantify this, we consider a type of noise called “large salt and pepper” on binary images. For this noise, we randomly add white and black squares of size $1\times 1$, $2\times 2$, $4\times 4$, and $8\times 8$ to the binary image. For instance, Fig. 9a illustrates an image contaminated by large salt noise.

Figure 9 also shows processed images using CNN with Median Layers and Algorithm 1. We observe in Fig. 9d–f that the CNN with Median Layers method fails to remove the larger features due to noise. This is most likely due to the CNN with Median Layer model being trained solely on images with typical, small scale salt and pepper noise and, therefore, that an expanded training set would be required for that approach to perform better on these images. On the other hand, Fig. 9g–i show denoised images produced by the unsupervised method given in Algorithm 1. We observe that these images are very clean and close to the ground truth. To further quantify their performances, similar to the experiment in “Experiments with salt and pepper noise” section, for each type of noise, we randomly generate 100 noised images and test the performance of these two algorithms by measuring IOU, PSNR, SSIM scores as well as calculating Betti numbers. The result is shown in Fig. 9j. We observe that Algorithm 1 performs better than CNN with Median Layers on this test set under all metrics.

## Conclusion

In this work, we establish that, under mild conditions, the morphological operations of erosion, dilation, opening, and closing may be combined to form multiparameter filtrations useful for studying binary images. These operations may also be combined with thresholding to form yet larger multiparameter filtrations useful for studying grayscale, and, by extension, color images. The dimension of the filtration grows with the number of operations and structuring elements, forming a potentially high dimensional framework in which to explore image structure and features. As demonstrated through the automated removal of salt and pepper and other small spatial scale noise from binary, grayscale, and color images in “Application: a denoising algorithm for salt and pepper noise” section, this framework can be used to create automated approaches to image analysis and processing. We believe that this work opens up a wide range of directions to pursue. We conclude this paper by mentioning a few of them. The experiment shows that the proposed algorithm outperforms classic algorithms on binary images that have salt and pepper noise, such as AMF and NAMF. On the other hand, although the CNN with median layers has better performance on erasing salt and pepper noise, the experiment in “Experiments with larger spatial scale noise” section shows that the proposed algorithm outperforms even CNN for larger spatial scale salt-and-pepper.

There is a much broader class of methods for extracting information from multiparameter filtrations than just the approach of calculating persistence along nondecreasing paths that we describe in Definition 8 and use in “Application: a denoising algorithm for salt and pepper noise” section. Persistent homology may be generalized as a cellular sheaf defined on a partially ordered set $(P,\le )$, that is, a functor from *P* to the category of vector spaces^58–61^. Cellular sheaves were originally developed for studying nerve theory in topology^62^ and have recently been used for describing the persistence of objects in applied topology. Because the order in Definition 5 is also a partial order on ${{\mathbb {Z}}}^k$, persistent homology defined on a multifiltration has a natural cellular sheaf structure. The persistence of the structure is much more complicated since the totally ordered property fails on the new order. However, we do see a variety of approaches for analyzing topological features in this setting, such as sheaf cohomology^59,63,64^, zig-zag homology^65^, multi-graded Betti numbers^25^, and rank invariants^24^. The multiparameter filtration we create here offers a constructive class of examples on which to explore these methods.

Recently, some studies have incorporated information from persistence diagrams into deep learning architecture (see e.g.^16,66–68^). Since the proposed (larger) multi-parameter filtration contains an even greater level of information about studied images, it would be interesting to incorporate such information into deep learning architecture. In addition, mathematical morphology, a classic topic in image processing, offers a wide range of operations for different image processing tasks. In this paper, we only consider four fundamental morphological operations. It will be interesting to investigate whether other types of morphological operations yield one/multi-parameter filtrations. For instance, an immediate application of this work is to form a bi-filtration by combining thresholding with the distance transform^69^, incorporating these operations into the existing framework to form a comprehensive, multi-parameter filtration. Lastly, as we mentioned in “New filtrations based on morphological operations” section, sequences of structuring elements satisfying the shift inclusion^37^ property also satisfy conditions (A1), (A2), and (A3) of Theorem 1 and can therefore also be used for constructing multi-parameter filtrations. In this paper, we mainly consider the sequence of $n \times n$ squares to approximate the geometry of local regions in images. Because different structuring elements can uncover different local geometric information, it will be interesting to consider other types of structuring elements (e.g. straight lines and discrete star shapes^13^) in building multi-parameter filtrations.

## Supplementary Information

Supplementary Information.

## Data Availability

No datasets were generated or analysed during the current study. Our codes are available on https://github.com/peterbillhu/MM_PersistentHomology.
