# Supplementary material for: A multi-parameter persistence framework for mathematical morphology
Source: Sci Rep. 2022 Apr 19;12:6427. doi: 10.1038/s41598-022-09464-7 (PMC9019063; doi:10.1038/s41598-022-09464-7)
Supplement: Supplementary file 1 — Supplementary Information. [file 41598_2022_9464_MOESM1_ESM.pdf]

Supplementary Information

Yu-Min Chung[1,*], Sarah Day[2], and Chuan-Shen Hu[3]

[1]Eli Lilly and Company, Indianapolis, Indiana 46225, USA
[2]Department of Mathematics, William & Mary, Williamsburg, Virginia 23185, USA
[3]Department of Mathematics, National Taiwan Normal University, Taipei City 106, Taiwan
[*]yumchung@alumni.iu.edu

ABSTRACT

This is the supplementary information for the paper "A Multi-parameter Persistence Framework for Mathematical Morphology". It provides the table of evaluation results of metrics IOU, β_0, β_1, PSNR, and SSIM in Figure 6 in the paper.

Density	Noised images	Algorithm 1	`denoiseImage`	NAMF	CNN with median layers
IOU					
0.1	0.8103 ± 0.0038	0.9809 ± 0.0031	0.9311 ± 0.0045	0.6211 ± 0.0048	0.9709 ± 0.0015
0.2	0.6694 ± 0.0036	0.9553 ± 0.0105	0.9332 ± 0.0043	0.5569 ± 0.0069	0.9608 ± 0.0017
0.3	0.5610 ± 0.0035	0.9131 ± 0.0197	0.8949 ± 0.0061	0.4787 ± 0.0114	0.9501 ± 0.0022
0.4	0.4734 ± 0.0035	0.8830 ± 0.0278	0.8087 ± 0.0070	0.3494 ± 0.0168	0.9404 ± 0.0022
0.5	0.4029 ± 0.0039	0.8351 ± 0.0177	0.6696 ± 0.0098	0.2026 ± 0.0231	0.9267 ± 0.0028
0.6	0.3445 ± 0.0029	0.7320 ± 0.0173	0.5094 ± 0.0083	0.1149 ± 0.0411	0.9056 ± 0.0040
0.7	0.2946 ± 0.0027	0.5786 ± 0.0171	0.3760 ± 0.0064	0.0970 ± 0.0914	0.8678 ± 0.0059
0.8	0.2523 ± 0.0025	0.4208 ± 0.0154	0.2854 ± 0.0046	0.0965 ± 0.1105	0.7803 ± 0.0100
0.9	0.2156 ± 0.0023	0.2962 ± 0.0103	0.2262 ± 0.0032	0.1079 ± 0.1129	0.4767 ± 0.0147
1.0	0.1837 ± 0.0022	0.2159 ± 0.0099	0.1835 ± 0.003	0.1005 ± 0.1097	0.2068 ± 0.0072
β_0					
0.1	246.50 ± 13.11	6.02 ± 0.14	35.03 ± 7.03	2.80 ± 1.35	6.00 ± 0.00
0.2	374.27 ± 15.21	6.02 ± 0.25	21.36 ± 3.88	2.04 ± 1.09	6.00 ± 0.00
0.3	413.23 ± 13.31	5.85 ± 0.64	33.44 ± 5.29	3.23 ± 1.45	6.00 ± 0.00
0.4	388.95 ± 15.39	5.66 ± 0.77	55.81 ± 7.03	5.73 ± 2.21	6.00 ± 0.00
0.5	319.27 ± 16.19	5.27 ± 0.83	80.74 ± 8.77	5.12 ± 2.35	6.00 ± 0.00
0.6	233.41 ± 16.03	5.34 ± 1.41	89.26 ± 9.66	4.28 ± 4.87	6.00 ± 0.00
0.7	159.84 ± 13.36	7.52 ± 2.08	75.08 ± 8.03	6.41 ± 7.61	6.08 ± 0.39
0.8	114.87 ± 12.41	17.33 ± 3.94	61.16 ± 8.67	4.28 ± 4.38	6.10 ± 0.76
0.9	106.57 ± 12.27	47.62 ± 6.59	62.6 ± 7.81	1.48 ± 0.99	16.72 ± 3.98
1.0	142.92 ± 14.56	70.27 ± 12.94	87.03 ± 11.59	1.01 ± 0.1	135.1 ± 12.97
β_1					
0.1	1153.17 ± 29.31	4.92 ± 0.34	302.6 ± 27.01	11.07 ± 3.23	5.00 ± 0.00
0.2	2057.41 ± 39.13	4.66 ± 0.77	147.63 ± 15.92	24.26 ± 4.77	5.00 ± 0.00
0.3	2699.55 ± 37.29	4.36 ± 1.13	172.78 ± 18.95	38.54 ± 7.14	5.00 ± 0.00
0.4	3125.99 ± 43.66	5.05 ± 1.51	326.50 ± 22.48	62.28 ± 10.12	4.96 ± 0.20
0.5	3341.63 ± 40.47	7.62 ± 2.42	661.47 ± 32.09	54.25 ± 9.53	4.88 ± 0.33
0.6	3376.46 ± 49.81	16.46 ± 4.06	1200.23 ± 40.39	20.67 ± 6.00	4.50 ± 0.61
0.7	3254.64 ± 50.98	23.91 ± 5.59	1767.64 ± 51.47	102.51 ± 562.70	3.89 ± 0.84
0.8	3015.38 ± 49.99	16.31 ± 3.75	2152.05 ± 54.89	602.12 ± 1208.84	7.95 ± 2.43
0.9	2679.09 ± 64.73	4.25 ± 2.05	2229.79 ± 66.2	1794.78 ± 1266.72	20.07 ± 4.48
1.0	2267.18 ± 58.03	0.41 ± 0.64	2012.95 ± 52.29	2154.61 ± 499.90	5.58 ± 2.84
PSNR					
0.1	0.3221 ± 0.0057	0.9645 ± 0.0048	0.6905 ± 0.0180	0.5340 ± 0.0021	0.9514 ± 0.0024
0.2	0.2231 ± 0.0026	0.9234 ± 0.0137	0.7683 ± 0.0170	0.5114 ± 0.0030	0.9353 ± 0.0028
0.3	0.1749 ± 0.0024	0.8621 ± 0.0250	0.6989 ± 0.0207	0.4840 ± 0.0057	0.9220 ± 0.0035
0.4	0.1393 ± 0.0024	0.8185 ± 0.0351	0.5232 ± 0.0180	0.4331 ± 0.0111	0.9046 ± 0.0035
0.5	0.1099 ± 0.0025	0.7533 ± 0.0234	0.3091 ± 0.0130	0.3875 ± 0.0201	0.8867 ± 0.0041
0.6	0.0843 ± 0.0019	0.6189 ± 0.0239	0.1601 ± 0.0062	0.3916 ± 0.0441	0.8634 ± 0.0048
0.7	0.0611 ± 0.0021	0.4232 ± 0.0245	0.0898 ± 0.0037	0.3953 ± 0.1070	0.8297 ± 0.0054
0.8	0.0400 ± 0.0021	0.2253 ± 0.0210	0.0514 ± 0.0030	0.3486 ± 0.1905	0.7653 ± 0.0076
0.9	0.0201 ± 0.0020	0.0896 ± 0.0104	0.0241 ± 0.0023	0.3066 ± 0.2168	0.4876 ± 0.0180
1.0	0.0016 ± 0.0017	0.0266 ± 0.0075	0.0019 ± 0.0019	0.3015 ± 0.2236	0.1001 ± 0.0086
SSIM					
0.1	13.0072 ± 0.0997	23.6458 ± 0.6791	17.8591 ± 0.3036	10.1089 ± 0.0508	21.8944 ± 0.2281
0.2	9.9958 ± 0.0659	19.9123 ± 0.966	18.0281 ± 0.2980	9.4000 ± 0.0617	20.5596 ± 0.1992
0.3	8.2477 ± 0.0500	16.8357 ± 1.0308	15.9551 ± 0.2779	8.6240 ± 0.1006	19.5564 ± 0.2053
0.4	6.9846 ± 0.0458	15.4218 ± 1.0292	13.0719 ± 0.1863	7.4225 ± 0.1356	18.6576 ± 0.1727
0.5	6.0210 ± 0.0503	13.6758 ± 0.5169	10.1618 ± 0.1693	6.3416 ± 0.1641	17.7052 ± 0.1779
0.6	5.2303 ± 0.0329	11.0626 ± 0.3912	7.6698 ± 0.1135	5.9121 ± 0.4389	16.5193 ± 0.2026
0.7	4.5599 ± 0.0292	8.1837 ± 0.2934	5.8028 ± 0.0785	5.4110 ± 1.3091	14.8823 ± 0.2246
0.8	3.9795 ± 0.0288	5.5864 ± 0.2573	4.5205 ± 0.0588	4.6362 ± 2.2466	12.2460 ± 0.2532
0.9	3.4655 ± 0.0223	3.4766 ± 0.1699	3.6531 ± 0.0337	4.1254 ± 2.5227	6.4400 ± 0.2600
1.0	3.0087 ± 0.0226	1.9912 ± 0.2851	3.0189 ± 0.0314	4.0690 ± 2.5714	1.7784 ± 0.0754

Table S1. The numerical values computed to produce Figure 7 in the main document. All scores are recorded by mean ± standard deviation for the 100 trials. The Betti pair (β_0, β_1) of the original image is $(6, 5)$.
